# Analysis of Spanish Radiometric Networks with the Novel Bias-Based Quality Control (BQC) Method

**DOI:** 10.3390/s19112483

**Published:** 2019-05-30

**Authors:** Ruben Urraca, Javier Antonanzas, Andres Sanz-Garcia, Francisco Javier Martinez-de-Pison

**Affiliations:** 1 EDMANS group, Department of Mechanical Engineering, University of La Rioja, C/San José de Calasanz, 31, 26004 Logroño, Spain; antonanzas.javier@gmail.com (J.A.); fjmartin@unirioja.es (F.J.M.-d.-P.); 2 Department of Mechanical Engineering, University of Salamanca, ETSII, Avda, Fernando Ballesteros, 2, 37700 Béjar, Spain; ansanz@usal.es; 3 Division of Pharmaceutical Biosciences, University of Helsinki, Viikinkaari 5 E (P.O. Box 56), 00014 Helsinki, Finland, Spain

**Keywords:** quality control, solar radiation, satellite-based data, reanalysis, pyranometer, weather stations, Spain

## Abstract

Different types of measuring errors can increase the uncertainty of solar radiation measurements, but most common quality control (QC) methods do not detect frequent defects such as shading or calibration errors due to their low magnitude. We recently presented a new procedure, the Bias-based Quality Control (BQC), that detects low-magnitude defects by analyzing the stability of the deviations between several independent radiation databases and measurements. In this study, we extend the validation of the BQC by analyzing the quality of all publicly available Spanish radiometric networks measuring global horizontal irradiance (9 networks, 732 stations). Similarly to our previous validation, the BQC found many defects such as shading, soiling, or calibration issues not detected by classical QC methods. The results questioned the quality of SIAR, Euskalmet, MeteoGalica, and SOS Rioja, as all of them presented defects in more than 40% of their stations. Those studies based on these networks should be interpreted cautiously. In contrast, the number of defects was below a 5% in BSRN, AEMET, MeteoNavarra, Meteocat, and SIAR Rioja, though the presence of defects in networks such as AEMET highlights the importance of QC even when using *a priori* reliable stations.

## 1. Introduction

Solar radiation measurements are essential for different applications such as solar resource assessment [1], climate trend analysis [2], and the estimation of meteorological and environmental variables [3]. The most widely recorded quantity is the global horizontal irradiance ($G_{H}$). However, even $G_{H}$ measurements are scarce due to the sparsity of stations with pyranometers. Besides, the temporal coverage of some of these stations is limited to the last 10–15 years. Therefore, several estimation methods have been developed to overcome the shortage of $G_{H}$ measurements [4,5]. Satellite-based models and atmospheric reanalysis are the most widely used techniques. Both provide estimations as solar radiation databases with almost global coverage up to the 1980s and spatio-temporal resolutions up to 1 km and 15-min. However, and despite the progress achieved by these models during the last decades [6], the uncertainty of the best satellite-based models is still higher than that of high-quality and well-maintained pyranometers [7]. Therefore, solar radiation measurements are not only fundamental to validate these databases [8,9] but they are also essential in those applications demanding low uncertainty data.

The aforementioned scarcity of solar radiation measurements makes them very valuable and some users take their quality for granted, especially when using reliable networks. However, different types of measuring errors can increase the uncertainty in solar radiation records. Measuring errors are broadly classified into equipment and operational defects [10]. Equipment errors originate from the limitations of pyranometers and constitute the inherent uncertainty of the sensor. They persist if the same sensor is installed at different sites. Some examples are cosine error, non-linearity, temperature dependence, or spectral error [11]. Equipment errors strongly depend on the type of detector (thermopile or photodiode) and the quality of the sensor, which is mainly related to its price, and their consequences can be aggravated if the pyranometer is not calibrated correctly. Operational errors can increase the uncertainty of measurements even further. They depend on the location of the sensors, the operation conditions (e.g., maintenance protocols), and the influence of external factors. Examples of operational errors include shadows by nearby objects, accumulation of dew, frost, snow or dust (soiling) on the sensors, incorrect leveling of the pyranometer, station shut-downs, and any failure in the ancillary equipment (data logger, sun trackers, data processing system), among others [10]. They can be prevented by selecting adequate sites, using high-quality equipment, and implementing strict maintenance protocols [12].

Several quality control (QC) methods have been proposed to detect measuring errors. Range tests such as the ones from the BSRN [13] are the most widely used. However, they cannot detect defects that introduce low-magnitude deviations such as shading, soiling, or calibration errors, because of the wide range of physically and statistically possible values of $G_{H}$. This is because solar radiation depends on stochastic processes such as cloud formation that can drastically alter the surface irradiance in just a few minutes. Consequently, the probability of including measuring errors in solar radiation studies is high even after applying range QC tests, especially when using non-reliable networks. To overcome this issue, we presented a new QC algorithm, the Bias-based Quality Control (BQC) [14], that is tailored for detecting low-magnitude defects. The BQC analyzes if the deviations between several independent radiation databases and the measurements are out of the typical range for that region and time of the year. Working with $G_{H}$ deviations instead of $G_{H}$ values allows narrowing the confidence intervals enabling the detection of low-magnitude defects. Besides, the method checks if a small deviation persists in a group of consecutive days, reducing the number of false alarms. The BQC was successfully validated at 313 European stations [15] finding different defects such as snow accumulation over the sensor, soiling, shading, and calibration drifts. Some of these defects were found in national meteorological services, reinforcing the importance of QC even when *a priori* reliable networks are used.

This paper further tests the BQC method by analyzing the quality of all publicly available Spanish weather stations measuring $G_{H}$. Our main goal is to provide potential users of solar radiation data with valuable information about the quality of the Spanish radiometric networks. Besides, we seek to extend the BQC validation by using a larger test dataset. Our previous validation was made with 313 European stations [15], mostly belonging to national meteorological services. In contrast, the current validation is made with 732 stations from 9 monitoring networks including both reliable networks, such as the national meteorological service (AEMET), and regional or agricultural networks whose quality may be more questionable. Thus, having a larger dataset and including secondary networks may allow us finding a wider variety of measuring errors to test the BQC method in depth.

## 2. Materials and Methods

### 2.1. Weather Stations

**Figure 1:**
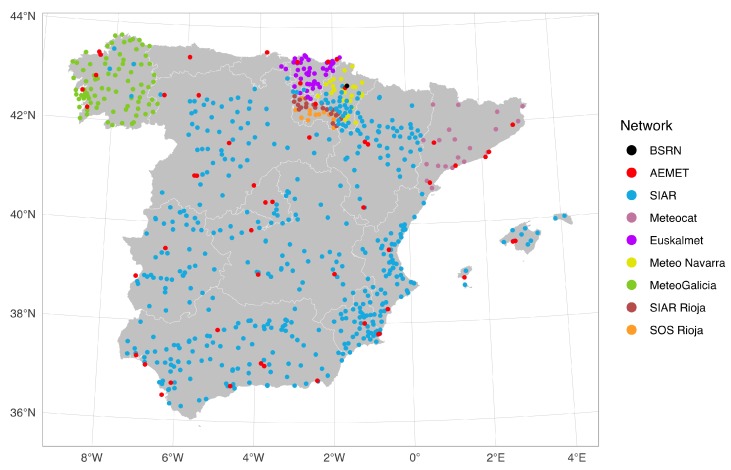
Locations of the weather stations used in the study.

$G_{H}$ measurements from all publicly available Spanish weather stations were retrieved from 2005 to 2013 at the highest temporal resolution freely provided (Figure 1). The resulting dataset comprised 732 stations from 9 monitoring networks (Table 1). The dataset included the BSRN station [16] located at Cener (CNR), Pamplona. The two national networks were the “Agencia Estatal de Meteorología” (AEMET) [17], which is the national meteorological service, and the “Servicio Integral de Asesoramiento al Regante” (SIAR) [18], a governmental network created for irrigation planning. Note that some SIAR stations belong to the Spanish Ministry of Agriculture and some others to the regional governments. Thus, some differences may exist in the maintenance and calibration protocols between stations. Most regional networks are the meteorological agencies of different Spanish regions: Meteo Navarra [19] (Navarra), Meteocat [20] (Cataluña), Euskalmet [21] (País Vaso), and MeteoGalicia [22] (Galicia). The remaining regional networks are SIAR Rioja [23], the SIAR branch at La Rioja, and SOS Rioja [24], the emergencies network of the Government of La Rioja.

Most meteorological networks use thermopile pyranometers (281 stations). They were classified according to the ISO 9060:1990 [25] from highest to lowest quality in (i) secondary standard; (ii) first class; and (iii) second class. On the other hand, photodiode pyranometers are the common sensor in agricultural networks (380 stations) due to its low price and the relatively low maintenance required. The description of the sensor was not provided in 71 stations. The complete list of the stations used in the study is available in Table S1.

### 2.2. Bias-Based Quality Control (BQC) Method

The BQC [14] is a semi-automatic method that combines model comparison and visual inspection techniques to detect low-magnitude errors in ground measurements. In short, the BQC analyzes groups of consecutive days with a window function flagging those groups in which the daily deviations of several independent radiation databases statistically differ from the typical values in that region and time of the year. All databases used must be temporally stable to achieve a proper characterization of the deviations. The BQC generates two color-coded plots to help in the inspection of the quality flags generated.

#### 2.2.1. Calculation of the Confidence Intervals (CIs)

The first step is to find the typical range of daily deviations for each radiation database in each region analyzed and time of the year. This is done statistically by defining a confidence interval (CI) within which the daily deviations of each radiation database lie. The daily deviations are the difference between estimations and measurements of the variable *X* being analyzed:

(1) $$deviation_{d}\left(X\right)=X_{d}^{est}-X_{d}^{mea}$$

The CIs are defined as the median absolute deviation ($\tilde{MAD}$) around the median bias deviation ($\tilde{MBD}$). They are calculated for each month of the year *m* (temporal averaging) and each spatial region sr sharing similar characteristics (spatial averaging):

(2) $$CI_{m,sr}^{db}={\tilde{MBD}}_{m,sr}^{db}\pm n\cdot {\tilde{MAD}}_{m,sr}^{db}\;m\in \left(Jan,\ldots ,Dec\right),\;sr\in spatial\;regions,\;db\in databases$$

where *n* is a tuning parameter that weights the $\tilde{MAD}$ in order to tune the restriction level of the QC method. The $\tilde{MAD}$ around the median has proven to be a more robust method for detecting outliers than the traditional standard deviation around the mean [26]. The $\tilde{MBD}$ and the $\tilde{MAD}$ are obtained in two steps to increase the robustness of the method. First the $\tilde{MBD}$ is calculated for all stations (st) and all months of the time series ($m^{\prime }$) as:

(3) $$\begin{array}{c} {\tilde{MBD}}_{m^{\prime },st}^{db}=median_{m^{\prime },st}^{db}\left(deviation_{d,st}^{db}\left(X\right)\right) \end{array}$$

These values are subsequently averaged again by grouping the months of the time series ($m^{\prime }$) in the twelve months of the year (*m*) (temporal averaging) and stations (st) in spatial regions (sr) (spatial averaging). This results in a unique set of twelve CIs per spatial group and radiation database:

(4) $$\begin{array}{c} \begin{array}{c} {\tilde{MBD}}_{m,sr}^{db}=median_{m,sr}^{db}\left({\tilde{MBD}}_{m^{\prime },st}^{db}\right)\\ {\tilde{MAD}}_{m,sr}^{db}=1.4826\cdot median_{m,sr}^{db}\left(\right|{\tilde{MBD}}_{m^{\prime },st}^{db}\left|\right) \end{array} \end{array}$$

The $\tilde{MAD}$ includes a constant scale factor of 1.4286 that ensures the consistency of estimates for different sample sizes (Equation (4)). The use of this constant value and the median makes this statistic more independent of the sample size and more robust than the standard deviation [26].

The CIs for each region should be calculated only with high-quality stations. The BQC can analyze any station within the regions defined, including those used to derive the CIs. If any defect is found in these stations, the CIs should be recalculated excluding those samples. Locations where radiation databases typically produce large deviations, such as snow-covered areas, small islands, or high mountains, should also be excluded from the calculation of the CIs. Besides, samples flagged at these locations should be examined carefully because the flags may be caused by deviations in the radiation database and not in the sensor (false alarm). If there are a sufficient number of stations where radiation databases show the same type of failure, they can be grouped in a specific spatial group.

#### 2.2.2. Flagging Samples with a Window Function

Having defined the CIs, a window function goes through the time series of each station flagging those groups of consecutive days where the daily deviations of all radiation databases are predominantly over or under the CI limits. The number of days analyzed by the window function each time is set with the window width (*w*) parameter. The distance between the first day of two consecutive windows is specified by the parameter step. Consecutive windows overlap because *w* is substantially larger than step.

**Figure 2:**
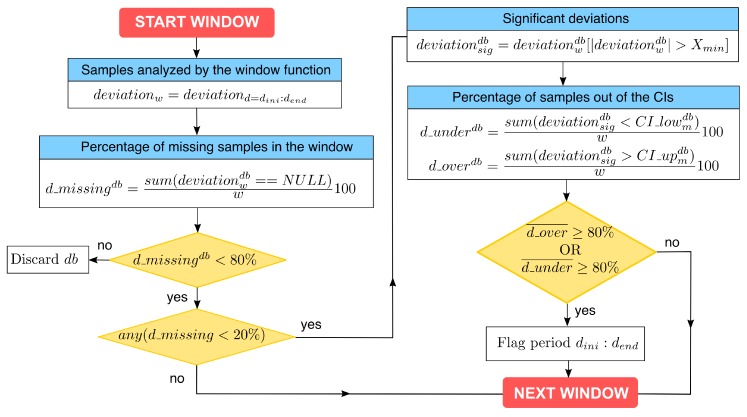
Flowchart of the window function (db = variable calculated for each radiation database, *d* = day of the time series, $d_{ini},d_{end}$ = first and last days of the time series, respectively, CI = confidence intervals of the daily deviations, CI_low, CI_up = lower and upper limits of the CI, *X* variable analyzed, $X_{min}$ minimum value of *X* to consider the deviations significant.)

Each analysis of the window function (Figure 2) starts with the calculation of the percentage of missing samples from each radiation database ($d\_missing^{db}$). Databases with more than 80% of missing values are discarded. Besides, at least one database should span almost the whole window ($d\_missing^{db}<20\%$) to ensure that the analysis covers most of the variability within the window. In the remaining databases, the percentage of days with deviations over ($d\_over^{db}$) or under ($d\_under^{db}$) the CI limits are calculated and subsequently averaged ($\bar{d\_over}$, $\bar{d\_under}$). These percentages are calculated only with significant deviations ($deviation_{sig}$) larger than a threshold $X_{min}$ to reduce the number of false alarms in cases with too narrow CIs (e.g., low irradiance months). All days within the window are flagged if more than 80% of the deviations are either over the CI upper limit ($\bar{d\_over}>80\%$) or under the CI lower limit ($\bar{d\_under}>80\%$).

#### 2.2.3. Visual Inspection of Flagged Samples

The BQC automatically generates two plots to facilitate the visual inspection of the quality flags generated: (a) the time series of the daily deviations from all radiation databases; and (b) the time series of instantaneous irradiance from the sensor and radiation databases with sub-daily temporal resolution overlapped. Both plots include color-coded flags that shade those days flagged by the window function (yellow/orange flags). Additionally, a grey flag shows periods with missing samples, and a red flag shows samples that had not passed the BSRN “Extremely rare limits” and “Physically possible limits” [27]. The BSRN tests can be only applied to measurements with sub-daily temporal resolution. Even though the window function works with daily means, it is convenient to include at least one database with sub-daily resolution to generate the plot of instantaneous $G_{H}$ because this usually allows finding the cause of the error.

### 2.3. Implementation

The BQC window function works with daily irradiance means, so all the weather stations with sub-daily data were aggregated to daily resolution. Hourly means were initially calculated at stations with sub-hourly resolution. In the case of 1-min data, 15-min averages were calculated if at least 5 min were available. Then hourly means were obtained if all four 15-min values were valid, following the procedure described in Roesch et al. [28]. In the case of resolutions between 5 min and 30 min, hourly means were directly calculated if all sub-hourly values were available. Finally, daily means were obtained by averaging all hourly values if at least 20 h were available.

The BQC was implemented with three independent databases: SARAH-1 [29], CLARA-A1 [30], and ERA-Interim [31]. SARAH-1 and CLARA-A1 are satellite-based databases produced by CM SAF from geostationary and polar-orbiting satellite images, respectively. ERA-Interim is a global reanalysis from the ECMWF. The solar radiation variability throughout Spain is small enough to filter out all the stations with the same CIs. The CIs were calculated using only AEMET stations, and they were recalculated eliminating those AEMET stations with measuring errors. The window function was run two times: (i) *w* = 20 d and *n* = 2.4; and (ii) *w* = 90 d and *n* = 0.4. In both cases, $X_{\min}$ was set to 5 $W/m^{2}$ or 5% to reduce the number of false alarms, and step was set to 5 d to seep up the whole process (fast-moving filter). This configuration was determined via grid search after analyzing different combinations of *w* an *n* [14]. The first run looks for short-lived defects analyzing windows of 20 d, relaxing the level of restriction of the CIs (*n* = 2.4) to reduce the number of false alarms. The second run seeks for long-lasting deviations using windows of 90 d. Here, the CIs can be made more restrictive (*n* = 0.4) in order to detect low-magnitude defects such as shading or calibration errors. Every flagged samples was visually inspected and classified into true defect or false alarm using the two plots generated by the BQC. Finally, true defects were classified into shading, soiling, snow/frost accumulation, time lags, diurnal values = 0, large errors, incorrect leveling, calibration errors, and unknown causes.

## 3. Results and Discussion

### 3.1. Analysis of the Quality of Monitoring Networks

**Figure 3:**
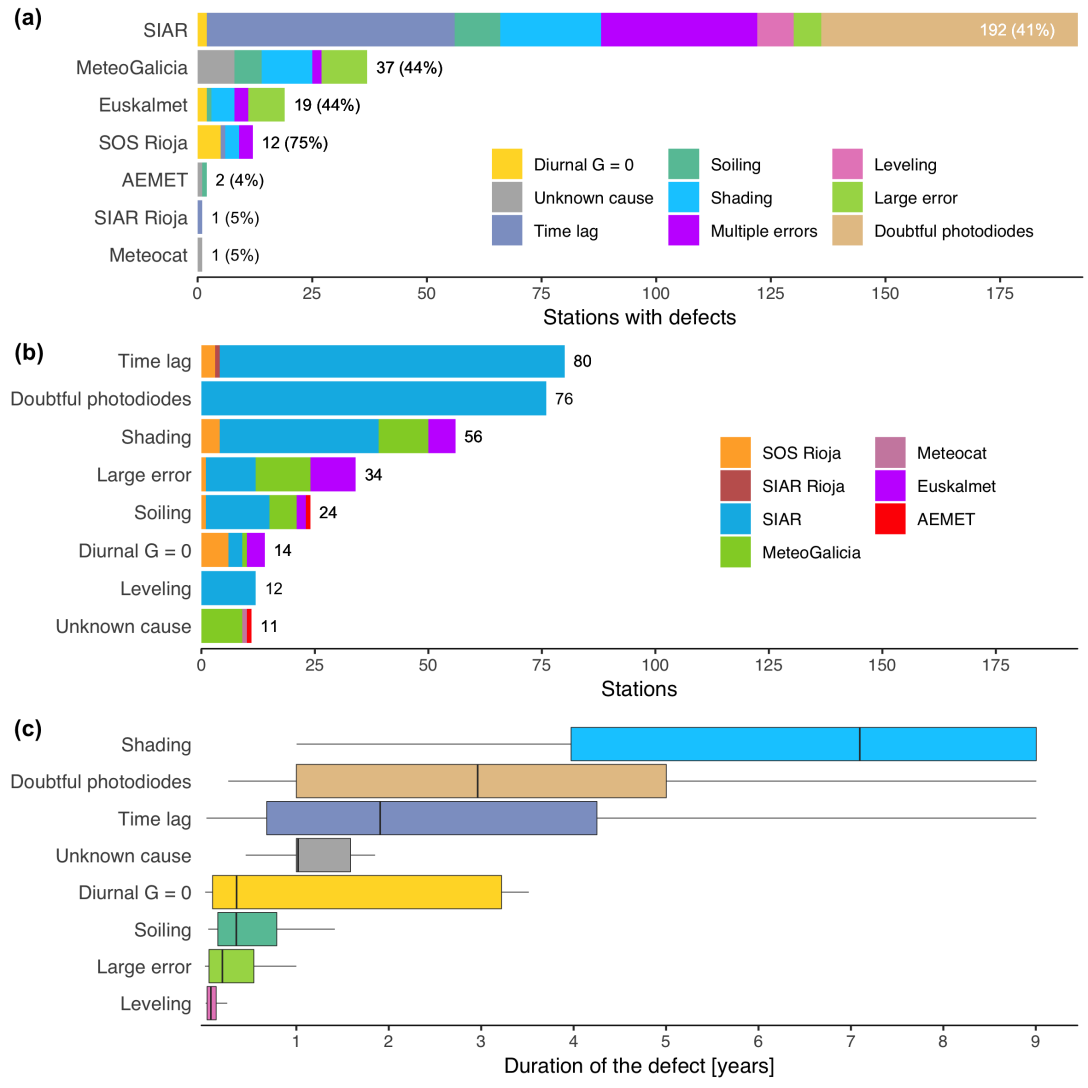
Summary of the defects found at the 732 weather stations. (**a**) Number of defects per network. The “multiple error” group includes those stations with two or more types of defects. The values in brackets show the percentage of stations with defects; (**b**) Number of stations per type of defect. Stations with multiple errors are accounted in all types of defects found; (**c**) Duration of each type of defect.

The BQC flagged suspect data at 264 out of the 732 stations (Figure 3). No false alarms were found after inspecting every flagged period. Most of the defects were solely detected with the BQC window function. The BSRN range tests, which were also integrated into the BQC, were only able to detect time lags, some cases of incorrect leveling, and some large errors such as positive $G_{H}$ values at night. However, the inclusion of the BSRN tests in the BQC is still very valuable because they enable to find defects such as time lags that cannot be detected in daily data. Most of the defects were operational errors, and hence most probably related to the inadequate maintenance of the stations. However, the BQC also flagged deviations at 76 doubtful SIAR stations probably related to photodiode limitations and their calibration.

**Figure 4:**
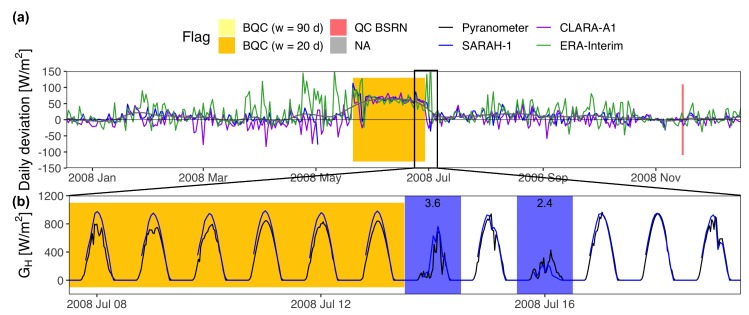
Accumulation of dust (soiling) over SIAR A12 pyranometer cleaned by the rain. (**a**) Daily deviations (estimations - measurements) of the radiation databases. The gray line is a smoother of the deviations from the three databases; (**b**) Instantaneous $G_{H}$ from SARAH-1 and the pyranometer. Days with precipitation are shaded in blue. The label shows the daily rainfall.

The majority of the defects were found in SIAR (166 stations, 35% of SIAR stations), partly because it is the most extensive network (468 stations). The Spanish Ministry of Agriculture created SIAR for irrigation planning, so most SIAR stations are located in agricultural regions such as Ebro and Guadalquivir Valleys or the Mediterranean Coast. Some stations were located close to other government facilities such as sewage-treatment plans to facilitate their maintenance. In contrast, pyranometers must be installed at locations with flat horizon and far from potential sources of contamination such as industrial areas, airports, or busy roads [12]. Thus, the inadequate location of some stations may explain the large number of stations with shadows (35 stations), or soiling (14 stations), due to the proximity of sources of pollution. Soiling is aggravated by the low maintenance and some pyranometers are not cleaned until it rains (Figure 4). The BSRN measuring guidelines [12] recommend cleaning the sensors at least once per day, preferably before dawn. Another indicator of the low maintenance in SIAR is the large number of stations with time lags (76 stations). Despite SIAR stations use UTC format, some of them lack for the daylight saving time correction during certain years. Besides, other variables such as temperature and precipitation are more critical than $G_{H}$ for agricultural purposes. The little interest on $G_{H}$ may also explain the large number of large errors, diurnal samples set to zero, or incorrectly leveled pyranometers. Most of these defects could have been prevented by implementing a basic QC protocol at the data processing center.

**Figure 5:**
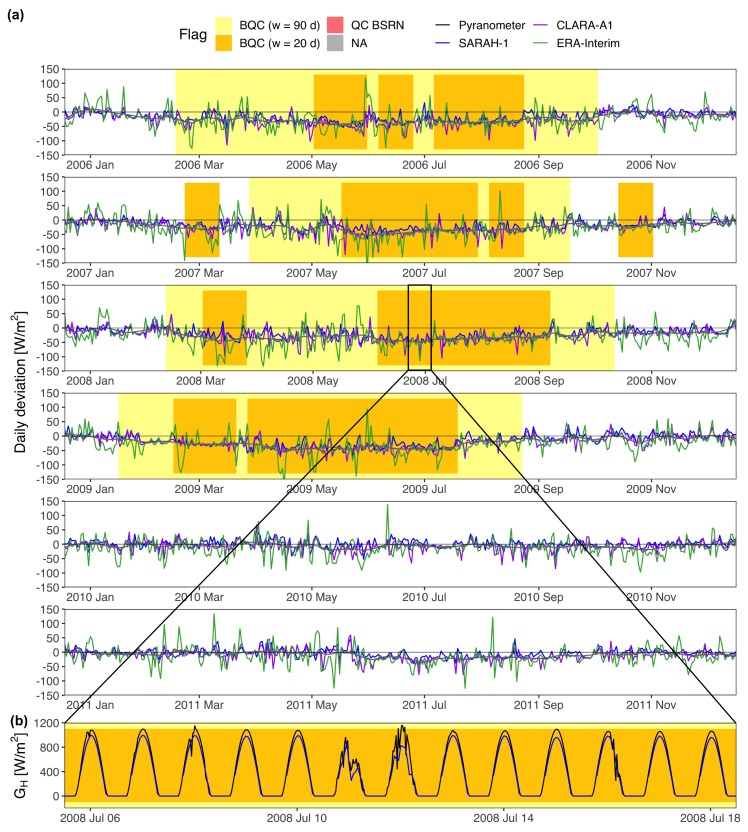
Incorrect photodiode calibration at SIAR ZA05 station. (**a**) Daily deviations (estimations-measurements) of the radiation databases. The gray line is a smoother of the deviations from the three databases; (**b**) Instantaneous $G_{H}$ from SARAH-1 and the pyranometer.

There were 76 additional SIAR photodiodes flagged by the BQC were we could not determine the exact type of operational error triggering the flag. The hypothesis of being false alarms was discarded because this type of flag only occurred in SIAR stations. In some cases, the BQC flagged a period of consecutive years with a constant bias that suddenly disappeared (Figure 5). The most likely explanation is the presence of a calibration bias during the first years of the time series that disappeared after a re-calibration. In other cases, the flags were at least 90 consecutive days of either positive or negative biases that randomly appeared through the time series. Although some of these flags could be caused by operational errors of low-magnitude, most of them may be triggered by the spectral response, cosine error, or temperature error of the photodiodes. Photodiodes are commonly installed in agricultural weather stations due to their low-cost, but their uncertainty can double that of thermopile pyranometers if they do not include adequate empirical corrections for the cosine error, spectral response, and linearity, among others [32]. This suggests that SIAR photodiodes are incorrectly calibrated, or calibrated with general empirical corrections without accounting for the specific conditions at the station. Besides, the calibration protocols may vary between stations because some SIAR stations are maintained by the Spanish Ministry while others belong to the regional governments. Overall, SIAR stations are inadequate to conduct solar radiation studies due to their high number of operational errors and the likely presence of equipment errors.

MeteoGalicia and Euskalmet are two regional meteorological agencies that provide high-resolution measurements (10-min) but present an unusually high number of defects for a meteorological network: 44% of Euskalmet stations, 44% of MeteoGalicia stations (Figure 3). This is especially alarming in Euskalmet because all stations are equipped with secondary standard pyranometers. The most common defect in both networks was large errors. Some of the defects identified, such as nocturnal periods with physically impossible values (Euskalmet), suggest that the QC protocols of both agencies are deficient. The Euskalmet QC protocol [33] is composed of five levels of quality flags that should have eliminated some of the large errors found in this study, such as the presence of large positive values at night. However, the data retrieved from http://opendata.euskadi.eus did not include any quality flag, suggesting that these data have not undergone the QC protocol described in Hérnandez et al. [33]. Shading and soiling are also frequent in both MeteoGalicia and Euskalmet, questioning the selection of some sites and the maintenance protocol. Similarly, Mirás-Avalos et al. [34] found several defects in MeteoGalicia and SIAR stations in Galicia with the QC procedure proposed by Younes et al. [10] with additional spatial consistency tests. They suggested that certain defects were caused by the handling of data, whereas others were attributed to shadows caused by nearby objects due to the bad positioning of the stations. Despite the high-quality expected *a priori*, $G_{H}$ measurements from both networks should be avoided.

**Figure 6:**
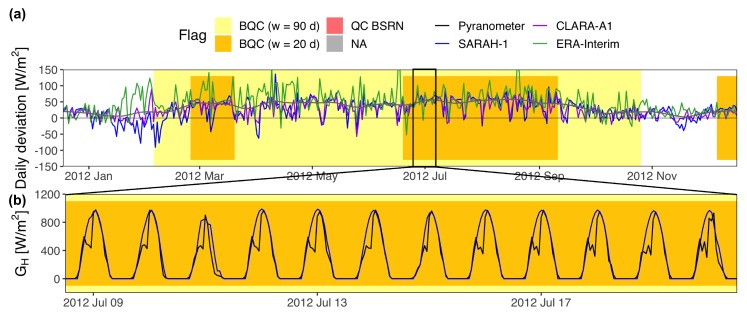
Shadows at SOS Rioja Urbaña station. (**a**) Daily deviations (estimations-measurements) of the radiation databases. The gray line is a smoother of the deviations from the three databases; (**b**) Instantaneous $G_{H}$ from SARAH-1 and the pyranometer.

SOS Rioja showed the worst quality overall as the 75% of their stations presented measuring errors. The most common one was the presence of diurnal periods with $G_{H}=0$ (6 stations). In some stations, this error appeared continuously during several years due to the lack of QC by the data processing center. Shading was another frequent defect in SOS Rioja (4 stations). Compared to other networks, the shadows occurred around solar noon (Figure 6) excluding the possibility of shadows being caused by obstacles on the horizon such as mountains, trees or buildings. As SOS Rioja sensors are installed in lattice towers, the most likely scenario is that the structure of the station is creating the shadows. This demonstrates deficient planning during the installation phase. In addition, the lack of maintenance and QC ruins the high quality of the first class pyranometers installed at all stations. Similarly to Euskalmet, these results corroborate that acquiring high-quality sensors does not guarantee obtaining high-quality records. Top-end pyranometers should be only installed if a proper maintenance program is implemented.

The number of defects in the other networks was low, with two defects in AEMET, one in SIAR Rioja and Meteocat, and no defects in Meteo Navarra and the BSRN. The good quality of the BSRN and AEMET was somehow expected. We recommend using these networks on applications requiring solar radiation data with low uncertainty. We also consider that Meteocat, Meteo Navarra, and SIAR Rioja present sufficient quality for being used in regional studies. Nevertheless, the presence of measuring errors in networks such as AEMET highlights the importance of implementing strict QC procedures even when reliable networks are used.

### 3.2. Influence of Measuring Errors in Solar Radiation Studies

The previous section showed that measuring errors are relatively common in regional and agricultural networks such as SIAR, Euskalmet, MeteoGalicia, and SOS Rioja. All of them presented defects in more than 40% of their stations (Figure 3a). Despite this, several studies have been published based on $G_{H}$ measurements from these networks, especially using SIAR stations [4,35,36,37,38,39,40,41,42,43,44,45]. SIAR is an attractive network for research studies in Spain because it freely provides 30-min $G_{H}$ data over a dense network of more than 500 stations covering most of Spain. On the contrary, studies based on AEMET stations are less common [7,46,47] because the access to AEMET sub-daily data is restricted. Weather stations from Euskalmet [33,48,49], MeteoGalicia [34], and SOS Rioja [50,51] have been also used to increase the density of solar radiation measurements over those regions. Therefore, it is very likely that the stations used in these studies contain some of the measuring errors found in the previous section.

The uncertainty of $G_{H}$ measurements may be particularly large in studies using SIAR stations because they not only may include operational defects but also doubtful photodiodes. Most of these studies assumed the calibration uncertainty of the photodiode used in SIAR stations (SP1110, Sky Instruments): an absolute accuracy of ±5% but typically lower than ±3%, for instantaneous $G_{H}$ [35,39,42,44]. Only Ruiz-Arias et al. [38] remarked that an uncertainty of ±7% may be a more realistic value. However, our previous comparison of SIAR photodiodes against AEMET secondary standards closer than 20 km [7] revealed that the real uncertainty of SIAR photodiodes is considerably larger (±15% daily $G_{H}$, ±5% yearly $G_{H}$). This value may be even larger at some stations because it was calculated after removing both operational defects and doubtful photodiodes. This comparison also revealed that SIAR photodiodes overestimated $G_{H}$ before 2010 by around +2% when compared to AEMET stations. A massive re-calibration of many photodiodes occurred around 2010 (e.g., station in Figure 5), but the bias did not completely disappear and most photodiodes still showed a negative deviation of around −1% compared to AEMET stations since 2010.

The quality control was deficient in the majority of the studies. It basically consisted of a two-step procedure: (i) discarding stations with too many missing values [38,39,40,41,42,44]; and (ii) discarding samples out of the range of physically possible values that were set based on the extraterrestrial irradiance [4,40,41], the clearness index [35,38], clear-sky models [42,45], or WMO recommendations [44]. However, range QC tests do not detect most of the errors present in the Spanish weather stations as shown in the previous section. In a different approach, Pagola et al. [36] selected the three closest SIAR stations to their goal location to discard the most divergent one. However, this approach may also fail due to the great percentage of defective SIAR stations. The most elaborated QC protocols were those used by Ruiz-Arias et al. [38] and Mirás-Avalos et al. [34]. Ruiz-Arias et al. [38] removed 14 SIAR stations in Andalucía after inspecting the data, observing suspicious inter-annual trends likely caused by deficient maintenance. Mirás-Avalos et al. [34] suggested that some of the defects they found using the QC method described in Younes et al. [10] were probably due to data handling errors and shading by nearby objects. Both were the only ones that questioned the quality of SIAR and MeteoGalicia data.

What is even worse, the QC was non-existent in some studies [37,43,48,49]. Some authors relied on the QC protocols implemented by the networks to justify the absence of QC in their studies. Almorox et al. [37] claimed that SIAR is responsible for the QC procedures that comprise the maintenance program of the network, including sensor calibration and data validation. However, the presence of defects such as diurnal records equal to zero demonstrates the lack of data validation by SIAR. Concerning the photodiodes, Ruiz-Arias et al. [42] stated that each SIAR station is subjected to biannual in situ inspections and that the radiation sensors are calibrated every year, while Rodriguez-Amigo et al. [44] stated that SIAR photodiodes are calibrated in accordance with ISO 9847. However, even if these calibration protocols were implemented, the results obtained in Urraca et al. [7] showed that the real uncertainty of SIAR photodiodes is larger than their calibration uncertainty. Besides, stations such as the one shown in Figure 5 confirm the presence of calibration errors. Concerning Euskalmet, Shiri et al. [48] affirmed that the data acquisition process and the quality procedures are the same for all studied stations. However, we verified in the previous section that the Euskalmet QC protocol [33] has not been implemented in the data available via http://opendata.euskadi.eus. As a consequence, most defects listed in Table 2 were probably not detected during the QC stage of the previous studies, so these defects may be affecting the results published.

Validations of radiation databases against SIAR stations are one of the best examples were measuring errors have great influence on the results [4,36,39]. The mean bias added by defects such as shading (−10%) [7] is substantially larger than that of the latest solar radiation databases (±0% to ± 8%) [46]. Besides, the uncertainty of incorrectly calibrated photodiodes can be similar to that of best satellite-based databases. These large uncertainties in solar radiation measurements prevent the accurate validation of the databases because deviations added by measuring errors may be mistaken for deviations of the model. The progress of solar modeling techniques is accentuating this problem because the magnitude of modeling errors is getting closer to that of operational defects. For instance, some of the errors obtained for the models of the following studies can be explained by the presence of measuring errors. Pagola et al. [36] compared several databases against five SIAR stations from 1999 to 2007. They obtained a negative bias in all stations that may be partly explained by the overestimation of SIAR photodiodes before 2010. Antonanzas-Torres et al. [39] validated a CM SAF SIS product against SIAR stations during 2010–2011 obtaining a bias of +3.41%. In this case, the large positive bias may be explained by the underestimation of SIAR photodiodes since 2010. Urraca et al. [4] validated SARAH-1 against SIAR stations in Castilla-La Mancha obtaining the largest positive bias at SIAR AB01 due to the shadows detected in the station (Table 2) and not due to SARAH-1 deficiencies. This type of abrupt variations in the bias distribution was our initial motivation to investigate the quality of SIAR stations and develop the BQC.

The consequences are even worse when $G_{H}$ measurements were used to correct the bias of radiation databases because the deviations caused by measuring errors were included in the bias-corrected databases [39,41,42]. For instance, Ruiz-Arias et al. [42] corrected the WRF NWP model with AEMET measurements (2003–2012), validating the corrected model against SIAR stations. The corrected database was free of measuring errors but these errors did affect the validation statistics. They observed an increasing bias in the corrected dataset that was attributed to the increasing number of AEMET stations after 2007. However, this trend also agrees with the decreasing bias observed in SIAR photodiodes from 2007 to 2013. The comparison of SIAR and AEMET stations in Urraca et al. [7] was made with a constant number of stations having data during all the years in the study period, so we believe that the trend observed by Ruiz-Arias et al. [42] was due to the high uncertainty and re-calibrations of SIAR photodiodes rather than to the increasing number of AEMET stations.

The consequences of using deficient weather stations were not so visible in the validation of locally-calibrated models such as empirical models [37,40,48,50], or forecasting models [49]. These models learn the patterns added by measuring errors, especially if the defect affects the whole time series. Thus, the effects of measuring errors are not observed when the model is validated in the same station where it was trained. However, they become visible when locally-calibrated models are benchmarked against satellite-based or NWP models. For instance, Antonanzas-Torres et al. [50] obtained a large positive bias at Urbaña and Moncalvillo (SOS Rioja) with a CM SAF database, which can be explained by the shadows found at those stations. Conversely, the empirical models showed moderate errors because they learned the shadows created by the own lattice of these stations.

Interpolation techniques are another type of locally-calibrated models that have exploited the high-density of SIAR stations [35,38,44]. The consequences of interpolating data with measuring errors may be visible in the irradiance maps obtained, which presented “spotty” distributions with sharp irradiance gradients around the stations. This was attributed to particular model configurations or the presence of anomalies in the explanatory variables [35]. However, systematic deviations of photodiodes or operational errors in SIAR stations may also contribute to these “spotty” distributions, and this possibility was not discussed in previous studies. The effects of measuring errors were more visible in the interpolation made by Rodriguez-Amigo et al. [44] in Castilla y León (2007–2013), because they trained the model with SIAR stations and validated it with four AEMET stations. In this case, the predominantly positive bias showed by most interpolation techniques evaluated agrees with the positive overall difference of +0.7% observed between SIAR and AEMET stations in 2007–20013.

Overall, the previous analysis showed that studies using SIAR, Euskalmet, MeteoGalicia, or SOS Rioja stations are not only common but also implement deficient QC procedures that are unable to detect the existing measuring errors. Besides, we have demonstrated the severity of the consequences of including measuring errors in some of these studies. This reinforces the importance of using solar radiation measurements with low uncertainty since this is the foundation where the conclusions of the study are built. The quality of the sensors and the maintenance protocols of the stations should always be checked. Besides, the use of advanced QC procedures such as the BQC method is strongly recommended to identify any operational or equipment failure, which can occur even in high-quality networks. Finally, the previous analysis also highlights the importance of making AEMET data publicly available at any temporal resolution without restrictions to improve the quality of studies using solar radiation data in Spain.

## 4. Conclusions

We analyzed the quality of all publicly available Spanish radiometric networks using the novel BQC method. The BQC detected a wide variety of measuring defects such as the presence of shadows, the accumulation of dust over the sensors, or calibration errors, which were not found by range QC methods. This allowed us to classify the Spanish monitoring networks according to their quality into two main groups. The first group of networks (SIAR, Euskalmet, MeteoGalicia, and SOS Rioja) presented operational and equipment defects in more than 40% of their stations. Hence, we recommend avoiding them for solar radiation studies in Spain. Otherwise, using elaborated QC methods such as the BQC is advisable due to the severe consequences observed in the studies that used stations from these networks but implemented deficient QC procedures. The second group (BSRN, AEMET, MeteoNavarra, SIAR Rioja, and Meteocat) presented defects in less than a 5% of their stations and are therefore adequate for solar radiation studies. However, the presence of measuring errors in networks such as AEMET reassures the importance of using elaborated QC procedures even when reliable stations are used.

## Figures and Tables

**Table 1:** Description of the monitoring networks used in the study. The values in brackets in the temporal resolution column are the interval midpoints. Types of pyranometers: secondary standard (SS), first class (FC), second class (SC), photodiode (Ph.), and not reported (NR).

			Types of Pyranometer					
Network	Type	Temporal Resolution	SS	FC	SC	Ph.	NR	Total
BSRN	radiometric	1 min	1	-	-	-	-	**1**
AEMET	meteorological	1 d	53	-	-	-	-	**53**
SIAR	agricultural	30 min (:00 UTC)	-	19	35	348	66	**468**
Meteo Navarra	meteorological	1 d	26	-	-	-	-	**26**
Meteocat	meteorological	1 d	-	15	-	5	-	**20**
Euskalmet	meteorological	10 min (:05 UTC)	43	-	-	-	-	**43**
MeteoGalicia	meteorological	10 min (:05 UTC)	1	21	34	27	1	**84**
SIAR Rioja	agricultural	1 h (:00 UTC)	-	-	21	-	-	**21**
SOS Rioja	emergencies	1 h (:00 UTC)	-	12	-	-	4	**16**
**Total**			**124**	**67**	**90**	**380**	**71**	**732**

**Table 2:** List of weather stations flagged by the BQC method.

Network	Type	Stations
AEMET	Soiling	5402
	Unknown cause	6325O
SIAR	Time lag	A09, A11, A16, AB07, AB08, AL01, AL05, AV01, AV101, AV102, BA01, BA07, BA104, BA205, BA207, BU102, CA07, CA10, CA101, CC04, CC09, CC102, CC13, CC16, CR01, CR02, CR03, CR10, CS11, GR09, GR10, H05, H06, H10, HU19, HU20, HU21, IB05, IB09, IB101, J102, J11, J15, LU02, M01, M102, MA09, MA10, MA101, NA09, NA101, NA102, NA103, NA104, NA105, NA106, NA107, NA108, NA109, NA110, NA111, SA03, SA101, SA102, SE101, SE12, SE13, SE17, TO10, TO11, TO12, V14, V26, VA06, VA102, ZA08
	Shading	A09, A10, A102, A1020, A13, AB01, AL10, BU101, CA05, CC10, CC14, CO04, CO08, CO102, GR03, GR09, GU07, IB04, IB07, IB08, IB09, J09, J102, MA09, MA10, MA101, V01, V101, V103, V104, V107, V19, V22, V24, VA05
	Soiling	A12, AL02, AL06, CA06, GR101, HU02, M01, MA06, MU03, MU11, TO03, TO09, V23, VA01
	Large error	A10, CR03, H101, J16, M04, MU10, MU17, NA14, SG01, V06, V104
	Diurnal $G_{H}$ = 0	J15, M05, VA101
	Leveling	A02, A07, A11, CC17, CS04, MA04, TE05, TO08, V01, V23, Z08, Z11
	Doubtful photodiode	A02, A03, A04, A08, A101, A11, AB01, AB02, AB05, AL08, AV101, BA101, BA102, BA203, C01, CC07, CC102, CC11, CC14, CC16, CO09, CR02, CS01, CS03, CS05, CS06, CS08, CS10, CS101, GR11, GU02, GU06, H01, H101, HU01, HU15, HU19, IB01, IB06, IB10, J01, J02, J03, J09, J12, LE01, M102, MA01, MA02, MA06, MU105, MU128, MU16, NA105, NA108, P02, P03, P07, SA03, SA101, SE02, SE08, SG02, V04, V05, V06, V07, V102, V14, V17, V20, V25, VA01, VA08, ZA05, ZA06
Meteocat	Unknown cause	DC
Euskalmet	Shading	023, 029, 051, 055, 058, 0DC
	Soiling	039, 047
	Large error	018, 026, 027, 030, 048, 054, 057, 060, 064, 0DC
	Diurnal $G_{H}$ = 0	020, 040, 047, 057
MGalicia	Shading	10052, 10053, 10057, 10060, 10063, 10064, 10086, 10088, 10095, 10108, 19065
	Soiling	10045, 10099, 10125, 10126, 19068, 19070
	Large error	10047, 10091, 10093, 10104, 10105, 10112, 10114, 10119, 10121, 10131, 10132, 10800
	Diurnal $G_{H}$ = 0	10105
	Unknown cause	10061, 10085, 10091, 10096, 10097, 10103, 10110, 10118, 10122
SIAR Rioja	Time lag	Albelda de Iregua
SOS Rioja	Time lag	Ezcaray, Santa Marina, Calahorra
	Shading	Urbaña, Moncalvillo, Calahorra, Villoslada
	Soiling	Ocón
	Large error	Ezcaray
	Diurnal $G_{H}$ = 0	Haro, Arnedo, Nájera, Ocón, Yerga, Torrecilla

## Supplementary Materials

The following are available online at https://www.mdpi.com/1424-8220/19/11/2483/s1, Table S1: List of weather stations.
