# Supplementary material for: Analysis of Spanish Radiometric Networks with the Novel Bias-Based Quality Control (BQC) Method
Source: Sensors (Basel). 2019 May 30;19(11):2483. doi: 10.3390/s19112483 (PMC6603785; doi:10.3390/s19112483)
Supplement: Supplementary file 1 [file sensors-19-02483-s001.pdf]

Supplementary Materials: List of weather stations

Ruben Urraca [1,*], Javier Antonanzas [1], Andres Sanz-Garcia [2,3] and Francisco Javier Martinez-de-Pison [1]

Table S1. List of the weather stations used in the study. Type of pyranometer (Pyr. type): secondary standard (SS), first class (FC), second class (SC), photodiode (Ph.), not reported (NR).

Network	ID	Name	Lon. [°]	Lat. [°]	Elev. [m]	Pyr. type
BSRN	CNR	Cener	-1.60	42.82	471	SS
AEMET	0016A	Reus/Aeropuerto	1.29	41.15	71	SS
AEMET	0201D	Barcelona CMT	2.20	41.39	6	SS
AEMET	1014A	San Sebastián/Fuenterrabia	-1.87	43.36	4	SS
AEMET	1024E	San SebastiánIgueldo	-2.11	43.31	251	SS
AEMET	1082	Bilbao/Aeropuerto	-2.96	43.30	42	SS
AEMET	1111	Santander ICMT	-3.81	43.49	52	SS
AEMET	1249X	Oviedo	-5.94	43.35	336	SS
AEMET	1387	A Coruña	-8.46	43.37	58	SS
AEMET	1387E	A Coruña/Alvedro	-8.42	43.31	98	SS
AEMET	1428	Santiago De Compostela/Labacolla	-8.51	42.89	370	SS
AEMET	1479I	Coron	-8.85	42.58	20	SS
AEMET	1495	Vigo/Peinador	-8.69	42.24	261	SS
AEMET	1549	Ponferrada	-6.60	42.56	534	SS
AEMET	2030	Soria	-2.63	41.77	1082	SS
AEMET	2422	Valladolid	-4.79	41.64	735	SS
AEMET	2462	NavacerradaPuerto	-4.11	40.79	1894	SS
AEMET	2661	León/Virgen Del Camino	-5.66	42.59	916	SS
AEMET	2867	Salamanca/Matacan	-5.63	40.96	790	SS
AEMET	2871D	Salamanca-Univ.	-5.70	40.96	800	SS
AEMET	3129	Madrid/Barajas	-3.61	40.47	609	SS
AEMET	3194U	Madrid C. Universitaria	-3.79	40.45	664	SS
AEMET	3260B	Toledo	-4.15	39.88	515	SS
AEMET	3469A	Cáceres	-6.39	39.47	394	SS
AEMET	367	Girona/Costa Brava	2.88	41.91	143	SS
AEMET	4121	Ciudad Real	-3.95	38.99	628	SS
AEMET	4478G	Badajoz (Universidad)	-7.13	38.88	175	SS
AEMET	4642E	Huelva Ronda Este	-7.02	37.28	19	SS
AEMET	5402	Córdoba/Aeropuerto	-4.97	37.85	90	SS
AEMET	5514	Granada/Base Aérea	-3.76	37.14	687	SS
AEMET	5530E	Granada/Aeropuerto	-3.85	37.19	567	SS
AEMET	5860E	El Arenosillo	-6.78	37.10	41	SS
AEMET	5960	Jerez de la Frontera/Aeropuerto	-6.11	36.75	27	SS
AEMET	5973	CádizObs.	-6.33	36.50	2	SS
AEMET	6156	Malaga (Centro Meteorologico)	-4.61	36.72	60	SS
AEMET	6325O	Almería/Aeropuerto	-2.42	36.85	21	SS
AEMET	7031	Murcia/San Javier	-0.83	37.79	4	SS
AEMET	7031X	Murcia/San Javier II	-0.86	37.78	4	SS
AEMET	7178I	Murcia	-1.21	38.00	61	SS
AEMET	76	Barcelona/Aeropuerto	2.10	41.29	4	SS
AEMET	8019	Alicante/El Altet	-0.61	38.28	43	SS
AEMET	8178D	AlbaceteObs.	-1.97	39.01	674	SS
AEMET	8368U	Teruel	-1.19	40.35	900	SS
AEMET	8414A	Valencia/Aeropuerto	-0.55	39.48	56	SS
AEMET	9091R	Vitoria/Foronda	-2.88	42.87	513	SS
AEMET	9170	Logroño/Agoncillo	-2.46	42.45	353	SS
AEMET	9263D	Pamplona/Noain	-1.65	42.78	459	SS
AEMET	9433	Zaragoza/Base Aérea	-1.13	41.68	252	SS
AEMET	9443R	Zaragoza (Argualas)	-1.05	41.63	250	SS
AEMET	9771C	Lleida	0.73	41.63	185	SS
AEMET	9981A	Tortosa	0.58	40.82	50	SS
AEMET	B228	Palma De Mallorca CMT	2.70	39.55	3	SS
AEMET	B278	Palma De Mallorca/Son San Juan	2.77	39.56	8	SS

AEMET	B954	Ibiza/Es Codolá	1.39	38.88	6	SS
SIAR	A02	Camp De Mirra	-0.77	38.68	627	Ph.
SIAR	A03	Vila Joiosa	-0.26	38.53	138	Ph.
SIAR	A04	Ondara	0.01	38.82	49	Ph.
SIAR	A05	Dénia Gata	0.08	38.79	102	Ph.
SIAR	A06	Pinoso	-1.06	38.43	606	Ph.
SIAR	A07	Novelda	-0.75	38.38	244	NR
SIAR	A08	Monforte del Cid	-0.73	38.40	244	Ph.
SIAR	A09	Crevillente	-0.78	38.24	94	Ph.
SIAR	A10	Almoradí	-0.77	38.03	74	Ph.
SIAR	A101	Elx Eea	-0.70	38.25	86	SC
SIAR	A102	Callosa d'en Sarrià	-0.10	38.65	160	Ph.
SIAR	A1020	Callosa d'en Sarrià (Malla)	-0.10	38.65	160	Ph.
SIAR	A11	Orihuela la Murada	-0.95	38.18	99	NR
SIAR	A12	Pilar de la Horadada	-0.81	37.87	77	Ph.
SIAR	A13	Catral	-0.81	38.15	27	Ph.
SIAR	A15	Altea	-0.08	38.60	210	Ph.
SIAR	A16	Castalla	-0.67	38.60	708	Ph.
SIAR	A18	Planes	-0.35	38.78	519	Ph.
SIAR	A19	Villena	-0.88	38.60	495	Ph.
SIAR	A20	Agost	-0.65	38.42	345	Ph.
SIAR	AB01	Tarazona	-1.92	39.26	722	Ph.
SIAR	AB02	Juanaco	-2.66	39.27	677	Ph.
SIAR	AB03	Almansa	-1.11	38.90	698	Ph.
SIAR	AB04	Hellín	-1.71	38.49	579	NR
SIAR	AB05	Albacete	-1.90	38.95	677	Ph.
SIAR	AB06	Ontur	-1.50	38.62	682	Ph.
SIAR	AB07	Pozo Cañada	-1.75	38.80	872	Ph.
SIAR	AB08	La Gineta	-2.01	39.10	722	Ph.
SIAR	AB09	Motilleja	-1.77	39.16	695	Ph.
SIAR	AB10	Caudete	-0.98	38.73	531	Ph.
SIAR	AL01	La Mojonera	-2.70	36.79	142	Ph.
SIAR	AL02	Almería	-2.40	36.84	22	Ph.
SIAR	AL04	Tabernas	-2.30	37.09	435	Ph.
SIAR	AL05	Fiñana	-2.84	37.16	971	Ph.
SIAR	AL06	Virgen de Fátima-Cuevas de Almanzora	-1.77	37.39	185	Ph.
SIAR	AL07	Huércal-Overa	-1.88	37.41	317	Ph.
SIAR	AL08	Cuevas de Almanzora	-1.80	37.26	20	Ph.
SIAR	AL10	Adra	-2.99	36.75	42	Ph.
SIAR	AL11	Níjar	-2.16	36.95	182	Ph.
SIAR	AL12	Tíjola	-2.46	37.38	796	Ph.
SIAR	AV01	Nava de Arévalo	-4.78	40.98	921	Ph.
SIAR	AV101	Muñogalindo	-4.93	40.58	1128	NR
SIAR	AV102	Losar del Barco	-5.53	40.37	1024	NR
SIAR	BA01	Zalamea de la Serena	-5.69	38.68	459	Ph.
SIAR	BA02	Monterrubio de La Serena	-5.38	38.59	499	Ph.
SIAR	BA03	Don Benito	-5.91	38.93	263	NR
SIAR	BA04	Villagonzalo	-6.19	38.84	266	Ph.
SIAR	BA05	Jerez de los Caballeros	-6.74	38.28	261	Ph.
SIAR	BA06	Olivenza	-7.06	38.72	202	Ph.
SIAR	BA07	Villafranca De Los Barros	-6.35	38.58	438	NR
SIAR	BA08	Don Benito	-5.90	38.93	267	Ph.
SIAR	BA09	Villafranca De Los Barros	-6.35	38.58	406	Ph.
SIAR	BA101	Mérida	-6.32	38.85	265	NR
SIAR	BA102	Azuaga	-5.71	38.39	540	SC
SIAR	BA103	Puebla de Alcocer	-5.10	39.07	500	SC
SIAR	BA104	Fuente de Cantos	-6.31	38.21	600	SC
SIAR	BA105	Don Benito-Efa	-5.86	38.98	260	SC
SIAR	BA106	Santa Amalia	-5.99	39.01	248	SC
SIAR	BA201	Acevedos	-7.08	38.77	209	NR
SIAR	BA202	Sagrajas	-6.91	38.91	174	NR
SIAR	BA203	Bercial	-6.83	38.88	188	SC
SIAR	BA204	Rueda Chica	-6.73	38.91	189	SC

SIAR	BA205	La Orden	-6.67	38.86	188	SC
SIAR	BA206	Arroyo	-6.47	38.86	221	SC
SIAR	BA207	Don Rodrigo	-6.35	38.66	362	NR
SIAR	BA209	Don Benito-Efa	-5.86	38.99	260	NR
SIAR	BA210	Palazuelo	-5.74	39.10	269	SC
SIAR	BU01	Miranda de Ebro	-2.97	42.69	479	NR
SIAR	BU02	Valle de Valdelucio	-4.13	42.75	975	Ph.
SIAR	BU03	Lerma	-3.77	42.04	840	Ph.
SIAR	BU04	Tardajos	-3.80	42.35	770	Ph.
SIAR	BU05	Vadocondes	-3.58	41.64	870	Ph.
SIAR	BU07	Santa Gadea del Cid	-3.08	42.70	532	Ph.
SIAR	BU101	Valle de Losa	-3.24	42.97	635	NR
SIAR	BU102	Condado de Treviño	-2.78	42.74	562	NR
SIAR	C01	A Capela	-8.05	43.45	372	Ph.
SIAR	C02	Boimorto	-8.14	43.03	429	Ph.
SIAR	CA01	Basurta-Jerez de la Frontera	-6.02	36.76	60	Ph.
SIAR	CA02	Jerez de la Frontera	-6.01	36.64	32	Ph.
SIAR	CA04	Villamartín	-5.62	36.84	171	Ph.
SIAR	CA05	Conil de la Frontera	-6.13	36.33	24	Ph.
SIAR	CA06	Vejer de la Frontera	-5.84	36.29	24	Ph.
SIAR	CA07	Jimena de la Frontera	-5.38	36.41	53	Ph.
SIAR	CA08	Puerto de santa María	-6.15	36.62	20	SC
SIAR	CA09	Sanlúcar de Barrameda	-6.31	36.78	22	SC
SIAR	CA10	Puerto de santa María	-6.16	36.61	9	Ph.
SIAR	CA101	IFAPA Centro de Chipiona	-6.40	36.75	7	SC
SIAR	CA11	Sanlúcar de Barrameda	-6.33	36.72	32	Ph.
SIAR	CC01	Casatejada	-5.68	39.87	274	Ph.
SIAR	CC03	Zarza De Granadilla	-6.05	40.21	383	NR
SIAR	CC04	Aldehuela del Jerte	-6.23	40.01	262	Ph.
SIAR	CC05	Moraleja	-6.68	40.05	252	NR
SIAR	CC07	Madrigalejo	-5.60	39.14	297	Ph.
SIAR	CC08	Valdesalor	-6.48	39.38	376	NR
SIAR	CC09	Peraleda de la Mata	-5.46	39.86	299	Ph.
SIAR	CC10	Valdeiñigos-Tejeda de Tíetar	-5.86	39.96	288	Ph.
SIAR	CC101	Hurdes-Azabal	-6.31	40.30	480	SC
SIAR	CC102	Madroñera	-5.76	39.46	625	SC
SIAR	CC103	Guadalupe	-5.35	39.39	740	SC
SIAR	CC104	Alcantara	-6.90	39.75	327	SC
SIAR	CC105	Mirabel	-6.21	39.85	541	NR
SIAR	CC106	Aliseda	-6.63	39.27	327	SC
SIAR	CC11	Jarandilla de la Vera	-5.65	40.10	508	Ph.
SIAR	CC12	Gargantilla	-5.94	40.24	596	Ph.
SIAR	CC13	Talayuela	-5.56	40.01	318	Ph.
SIAR	CC14	Coria-Puebla de Argeme	-6.46	39.96	235	Ph.
SIAR	CC15	Valdastillas	-5.88	40.14	475	NR
SIAR	CC16	Moraleja	-6.69	40.07	273	Ph.
SIAR	CC17	Valdastillas	-5.87	40.14	495	Ph.
SIAR	CC18	Valdesalor	-6.33	39.37	382	Ph.
SIAR	CC19	Zarza De Granadilla	-6.03	40.21	380	Ph.
SIAR	CO01	Bélmez	-5.21	38.25	523	Ph.
SIAR	CO02	Adamuz	-4.45	38.00	90	Ph.
SIAR	CO03	Palma del Río	-5.23	37.73	134	NR
SIAR	CO04	Hornachuelos	-5.16	37.72	157	Ph.
SIAR	CO05	El Carpio	-4.50	37.91	165	Ph.
SIAR	CO06	Córdoba	-4.80	37.86	117	Ph.
SIAR	CO07	Santaella	-4.89	37.52	207	Ph.
SIAR	CO08	Baena	-4.31	37.69	334	Ph.
SIAR	CO09	Palma del Río	-5.23	37.73	55	Ph.
SIAR	CO101	IFAPA Centro de Cabra	-4.43	37.50	547	SC
SIAR	CO102	IFAPA Centro de Hinojosa del Duque	-5.12	38.50	543	SC
SIAR	CR01	Alcázar de San Juan	-3.20	39.21	658	Ph.
SIAR	CR02	Manzanares	-3.36	39.10	648	NR
SIAR	CR03	Porzuna	-4.23	39.23	615	Ph.

SIAR	CR04	Ciudad Real	-3.99	39.04	662	Ph.
SIAR	CR05	Bolaños	-3.62	38.95	710	Ph.
SIAR	CR06	Villahermosa-Tajoneras	-2.80	38.84	966	NR
SIAR	CR07	Argamasilla de Alba	-3.06	39.08	727	Ph.
SIAR	CR08	Herencia	-3.33	39.38	659	Ph.
SIAR	CR09	Daimiel	-3.69	39.01	622	Ph.
SIAR	CR10	Manzanares	-3.35	39.12	652	Ph.
SIAR	CR101	Entresierra	-3.95	39.03	640	NR
SIAR	CR11	Montiel	-2.88	38.70	887	Ph.
SIAR	CS01	'Vall d"uixó'	-0.23	39.80	100	Ph.
SIAR	CS03	San Rafael del Río	0.37	40.59	205	Ph.
SIAR	CS04	Benicarló	0.40	40.41	12	Ph.
SIAR	CS05	Castelló Benadresa	-0.12	39.99	95	Ph.
SIAR	CS06	Burriana	-0.11	39.89	98	Ph.
SIAR	CS07	Ribera de Cabanes	0.15	40.13	34	Ph.
SIAR	CS08	Nules	-0.17	39.88	49	Ph.
SIAR	CS09	Segorbe	-0.48	39.82	358	Ph.
SIAR	CS10	Onda	-0.21	39.97	185	NR
SIAR	CS101	Vila-Real Eea	-0.14	39.94	42	Ph.
SIAR	CS11	Onda	-0.19	39.97	138	Ph.
SIAR	CU01	El Sanchón	-2.30	39.36	698	Ph.
SIAR	CU02	Pedernoso	-2.76	39.47	720	Ph.
SIAR	CU03	El Picazo	-2.09	39.45	728	Ph.
SIAR	CU04	Barajas de Melo	-2.94	40.11	707	Ph.
SIAR	CU05	Cañete	-1.65	40.03	1053	Ph.
SIAR	CU07	Villaconejos de Trabaque	-2.32	40.41	810	Ph.
SIAR	CU08	Villanueva de la Jara	-1.85	39.40	820	Ph.
SIAR	CU09	Mariana	-2.14	40.15	940	Ph.
SIAR	GR01	Baza	-2.77	37.56	814	Ph.
SIAR	GR02	Puebla de Don Fadrique	-2.38	37.88	1110	Ph.
SIAR	GR03	Loja	-4.14	37.17	487	Ph.
SIAR	GR04	Pinos Puente	-3.77	37.26	594	NR
SIAR	GR05	Iznalloz	-3.55	37.42	935	Ph.
SIAR	GR06	Jerez del Marquesado	-3.15	37.19	1212	Ph.
SIAR	GR07	Cádiar	-3.18	36.92	950	Ph.
SIAR	GR08	Zafarraya	-4.15	36.99	905	Ph.
SIAR	GR09	Almuñecar	-3.68	36.74	49	NR
SIAR	GR10	Padul	-3.60	37.02	781	Ph.
SIAR	GR101	IFAPA Centro Camino del Purchil	-3.64	37.17	630	SC
SIAR	GR11	Almuñecar	-3.68	36.75	14	Ph.
SIAR	GU01	Finca El Serranillo: Vivero Forestal	-3.17	40.66	665	NR
SIAR	GU02	Jadraque	-2.95	40.93	808	Ph.
SIAR	GU03	Armuña de Tajuña	-3.01	40.53	759	Ph.
SIAR	GU04	Alovera	-3.25	40.59	664	NR
SIAR	GU05	Prados Redondos	-1.80	40.80	1129	Ph.
SIAR	GU06	Illana	-2.99	40.21	589	Ph.
SIAR	GU07	Finca El Serranillo	-3.17	40.66	644	NR
SIAR	GU08	Alovera	-3.25	40.59	632	NR
SIAR	GU09	Marchamalo	-3.21	40.68	692	Ph.
SIAR	H01	El Tojalillo-Gibraleón	-7.03	37.32	52	NR
SIAR	H02	Lepe	-7.24	37.30	74	Ph.
SIAR	H03	Gibraleón	-7.06	37.41	169	Ph.
SIAR	H04	Moguer	-6.79	37.15	87	Ph.
SIAR	H05	Niebla	-6.74	37.35	52	Ph.
SIAR	H06	Aroche	-6.94	37.96	299	Ph.
SIAR	H07	La Puebla de Guzmán	-7.25	37.55	288	Ph.
SIAR	H08	El Campillo	-6.60	37.66	406	Ph.
SIAR	H09	La Palma del Condado	-6.54	37.37	192	Ph.
SIAR	H10	Almonte	-6.48	37.15	18	Ph.
SIAR	H101	IFAPA Finca El Cebollar	-6.80	37.24	63	SC
SIAR	HU01	Valfarta	-0.15	41.53	352	Ph.
SIAR	HU02	Zaidín	0.29	41.64	175	Ph.
SIAR	HU03	Alcolea de Cinca	0.07	41.74	227	Ph.

SIAR	HU04	Tamarite de Litera	0.38	41.78	221	Ph.
SIAR	HU05	Lanaja	-0.34	41.79	368	Ph.
SIAR	HU06	Selgua	0.13	41.94	304	Ph.
SIAR	HU07	Barbastro	0.11	42.01	410	Ph.
SIAR	HU08	Sariñena	-0.18	41.77	283	Ph.
SIAR	HU09	Huesca	-0.38	42.11	420	Ph.
SIAR	HU10	Candasnos	0.09	41.46	320	Ph.
SIAR	HU11	Grañén	-0.36	41.94	312	Ph.
SIAR	HU12	Huerto	-0.14	41.95	415	Ph.
SIAR	HU13	Gurrea de Gállego	-0.73	41.99	363	Ph.
SIAR	HU14	Banastón	0.17	42.39	562	Ph.
SIAR	HU15	Alfántega	0.15	41.82	246	Ph.
SIAR	HU16	Santa Cilia de Jaca	-0.72	42.56	666	NR
SIAR	HU17	Fraga	0.35	41.50	81	Ph.
SIAR	HU18	Tardienta	-0.51	41.97	361	Ph.
SIAR	HU19	San Esteban de Litera	0.30	41.88	315	Ph.
SIAR	HU20	Monte Julia	0.23	41.74	274	Ph.
SIAR	HU21	Sodeto	-0.26	41.88	312	Ph.
SIAR	HU22	Santa Cilia de Jaca	-0.71	42.58	666	Ph.
SIAR	IB01	Santa Eulalia	1.44	39.01	130	Ph.
SIAR	IB02	Inca	2.94	39.68	42	Ph.
SIAR	IB03	Manacor	3.17	39.55	84	Ph.
SIAR	IB04	Son Ferriol	2.73	39.56	28	Ph.
SIAR	IB05	Felanitx	3.09	39.48	109	Ph.
SIAR	IB06	Sa Pobla	3.04	39.80	6	Ph.
SIAR	IB07	Sóller	2.71	39.78	44	NR
SIAR	IB08	Es Mercadal	4.10	40.00	41	Ph.
SIAR	IB09	Artá	3.36	39.70	114	Ph.
SIAR	IB10	Calviá	2.46	39.55	20	Ph.
SIAR	IB101	Ciutadella	3.85	39.97	21	NR
SIAR	IB11	′S″ Estany des Peix′	1.40	38.73	11	Ph.
SIAR	J01	Huesa	-3.06	37.75	793	Ph.
SIAR	J02	Pozo Alcón	-2.93	37.67	893	Ph.
SIAR	J03	San José de Los Propios	-3.23	37.86	509	Ph.
SIAR	J04	Sabiote	-3.24	38.08	822	Ph.
SIAR	J05	Torreblascopedro	-3.69	37.99	291	Ph.
SIAR	J06	Alcaudete	-4.08	37.58	645	Ph.
SIAR	J07	Mancha Real	-3.60	37.92	436	Ph.
SIAR	J08	Ubeda	-3.30	37.94	358	Ph.
SIAR	J09	Linares	-3.65	38.06	443	Ph.
SIAR	J10	Marmolejo	-4.13	38.06	208	NR
SIAR	J101	Torreperogil	-3.24	37.97	536	SC
SIAR	J102	Villacarrillo	-3.20	38.06	650	SC
SIAR	J103	Jódar	-3.33	37.88	488	SC
SIAR	J104	IFAPA Centro Mengibar	-3.79	37.94	292	SC
SIAR	J11	Chiclana de Segura	-3.00	38.30	510	Ph.
SIAR	J12	La Higuera De Arjona	-4.01	37.95	267	Ph.
SIAR	J14	Santo Tomé	-3.08	38.03	571	Ph.
SIAR	J15	Jaén	-3.77	37.89	299	Ph.
SIAR	J16	Marmolejo	-4.18	38.05	231	Ph.
SIAR	LE01	Carracedelo	-6.71	42.57	480	Ph.
SIAR	LE02	Mansilla Mayor	-5.43	42.51	791	Ph.
SIAR	LE03	Cubillas de Los Oteros	-5.51	42.40	769	Ph.
SIAR	LE04	Zotes del Páramo	-5.74	42.26	779	Ph.
SIAR	LE05	Quintana del Marco	-5.84	42.22	750	Ph.
SIAR	LE06	Hospital de Orbigo	-5.90	42.46	835	Ph.
SIAR	LE07	Bustillo del Páramo	-5.77	42.46	874	Ph.
SIAR	LE08	Sahagún	-5.02	42.37	856	Ph.
SIAR	LE09	Santas Martas	-5.26	42.44	885	Ph.
SIAR	LU01	Castro de Rei	-7.49	43.16	414	Ph.
SIAR	LU02	Monforte de Lemos	-7.50	42.51	340	Ph.
SIAR	M01	Center: Finca Experimental	-3.50	40.41	604	Ph.
SIAR	M02	Arganda	-3.50	40.31	537	Ph.

SIAR	M03	Aranjuez	-3.63	40.04	487	Ph.
SIAR	M04	Fuentidueña de Tajo	-3.18	40.11	561	Ph.
SIAR	M05	San Martín de la Vega	-3.56	40.23	512	Ph.
SIAR	M06	Chinchón	-3.47	40.19	535	Ph.
SIAR	M102	Villa del Prado	-4.27	40.25	477	NR
SIAR	MA01	Málaga	-4.54	36.76	68	Ph.
SIAR	MA02	Vélez-Málaga	-4.13	36.80	49	Ph.
SIAR	MA03	Antequera	-4.56	37.06	457	NR
SIAR	MA04	Estepona	-5.21	36.44	199	Ph.
SIAR	MA05	Archidona	-4.43	37.08	516	NR
SIAR	MA06	Sierra Yeguas	-4.84	37.14	464	Ph.
SIAR	MA07	IFAPA Churriana	-4.50	36.67	32	Ph.
SIAR	MA08	Pizarra	-4.72	36.77	84	Ph.
SIAR	MA09	Cártama	-4.68	36.72	95	Ph.
SIAR	MA10	Antequera	-4.56	37.03	457	Ph.
SIAR	MA101	IFAPA Centro De Campanillas	-4.56	36.73	60	SC
SIAR	MU01	Finca Experimental De Aguilas (Cida)	-1.59	37.42	65	Ph.
SIAR	MU02	Finca Experimental De Totana (Cida)	-1.51	37.73	266	Ph.
SIAR	MU03	Forja S.a.-Fuente Alamo	-1.13	37.75	150	Ph.
SIAR	MU04	Los Martínez Del Puerto	-1.05	37.80	124	NR
SIAR	MU06	La Aljorra	-1.07	37.68	70	Ph.
SIAR	MU07	Roche. Finca La Torreta	-0.92	37.63	130	NR
SIAR	MU08	Turilla: La Paca	-1.82	37.86	713	Ph.
SIAR	MU09	Purias-Lorca	-1.63	37.60	441	Ph.
SIAR	MU10	Charco De Taray	-1.15	38.16	215	Ph.
SIAR	MU101	Alhama (La Calavera)	-1.42	37.79	168	FC
SIAR	MU102	Calasparra (Rotas)	-1.69	38.25	276	FC
SIAR	MU103	Molina De Segura (Campotejar)	-1.22	38.13	146	FC
SIAR	MU104	Murcia (Cabezo Plata)	-0.98	37.98	128	FC
SIAR	MU105	Mazarrón (Cañada Gallego)	-1.36	37.57	94	Ph.
SIAR	MU106	Cehegín (La Torrecita)	-1.78	38.10	511	FC
SIAR	MU107	Fuente Alamo (Campillo Bajo)	-1.24	37.70	175	FC
SIAR	MU108	Cehegín (El Chaparral)	-1.68	38.11	431	FC
SIAR	MU109	Puerto Lumbreras (El Esparragal)	-1.73	37.59	318	FC
SIAR	MU11	Finca Taray (Torres De Cotillas)	-1.30	38.01	169	Ph.
SIAR	MU110	Blanca (Estación De Blanca)	-1.31	38.24	280	FC
SIAR	MU111	Cieza (La Carrichosa)	-1.50	38.29	253	Ph.
SIAR	MU112	Jumilla (Las Encebras)	-1.24	38.39	400	FC
SIAR	MU114	Yecla (Pinillos)	-1.11	38.56	567	FC
SIAR	MU115	Jumilla (C^o del Judio)	-1.42	38.39	395	FC
SIAR	MU117	Torre Pacheco (Torre Blanca)	-0.90	37.77	32	FC
SIAR	MU119	Torre Pacheco (Los Infiernos)	-0.93	37.82	90	FC
SIAR	MU12	Villanueva De Segura	-1.33	38.13	173	NR
SIAR	MU120	San Javier (Santiago De La Ribera)	-0.82	37.79	6	FC
SIAR	MU121	Murcia (La Alberca)	-1.13	37.94	54	FC
SIAR	MU123	Mula (Mula)	-1.47	38.04	275	Ph.
SIAR	MU124	Caravaca (Barranda)	-1.99	38.04	867	FC
SIAR	MU125	Jumilla (Roman)	-1.33	38.32	340	FC
SIAR	MU126	Abanilla (La Jaira)	-1.05	38.17	140	FC
SIAR	MU128	Torre Pacheco (Torre Pacheco)	-0.99	37.75	56	Ph.
SIAR	MU130	San Javier (El Mirador)	-0.88	37.85	93	Ph.
SIAR	MU14	Finca Venta De Ulea-Moratalla	-1.81	38.20	515	Ph.
SIAR	MU15	Librilla	-1.34	37.90	299	Ph.
SIAR	MU16	Puerto Lumbreras	-1.69	37.50	382	Ph.
SIAR	MU17	Beniel	-1.00	38.03	56	Ph.
SIAR	MU18	Yecla	-1.19	38.66	661	Ph.
SIAR	MU19	Corvera	-1.12	37.83	237	Ph.
SIAR	MU20	Casa Rosa De Ulea	-1.26	38.19	240	Ph.
SIAR	NA01	22 Tudela	-1.57	42.07	258	Ph.
SIAR	NA02	23 Fitero	-1.84	42.05	450	Ph.
SIAR	NA03	24 Cascante	-1.72	42.03	330	Ph.
SIAR	NA04	25 Ablitas	-1.64	42.00	338	Ph.
SIAR	NA05	06 Aibar	-1.32	42.56	421	Ph.

SIAR	NA06	07 San Martín de Unx	-1.61	42.51	447	Ph.
SIAR	NA07	13 Murillo El Fruto	-1.49	42.38	393	Ph.
SIAR	NA08	02 Adiós	-1.75	42.69	444	Ph.
SIAR	NA09	05 Artajona	-1.79	42.58	353	Ph.
SIAR	NA10	08 Miranda de Arga	-1.81	42.51	343	Ph.
SIAR	NA101	20 Cadreita	-1.72	42.21	267	Ph.
SIAR	NA102	19 Bardenas Reales (Barranco)	-1.66	42.26	298	Ph.
SIAR	NA103	15 Sartaguda	-2.05	42.36	307	Ph.
SIAR	NA104	11 Olite	-1.66	42.42	397	Ph.
SIAR	NA105	14 Traibuenas	-1.62	42.36	312	Ph.
SIAR	NA106	01 Arazuri	-1.72	42.81	396	Ph.
SIAR	NA107	03 Lumbier	-1.28	42.67	484	Ph.
SIAR	NA108	04 Ancin	-2.17	42.66	469	Ph.
SIAR	NA109	21 Corella	-1.84	42.12	392	Ph.
SIAR	NA11	12 Falces	-1.79	42.41	292	Ph.
SIAR	NA110	18 Funes	-1.81	42.29	391	Ph.
SIAR	NA111	09 Lerin	-1.98	42.50	354	Ph.
SIAR	NA12	16 San Adrián	-1.89	42.34	385	Ph.
SIAR	NA13	10 Bargota	-2.30	42.48	375	Ph.
SIAR	NA14	17 Bardenas Reales (El Plano)	-1.52	42.30	425	Ph.
SIAR	NA15	26 Los Arcos	-2.18	42.54	419	Ph.
SIAR	NA16	27 Sesma	-2.13	42.47	438	Ph.
SIAR	NA17	San Adrián	-1.90	42.36	382	Ph.
SIAR	P01	Torquemada	-4.30	42.05	868	Ph.
SIAR	P02	Villamuriel de Cerrato	-4.49	41.95	750	Ph.
SIAR	P03	Fuentes de Nava	-4.72	42.08	744	Ph.
SIAR	P04	Villoldo	-4.59	42.27	817	Ph.
SIAR	P05	Lantadilla	-4.28	42.35	798	NR
SIAR	P06	Herrera de Pisuerga	-4.25	42.49	821	Ph.
SIAR	P07	Villaluenga de la Vega	-4.77	42.53	927	Ph.
SIAR	P08	Lantadilla	-4.28	42.35	798	Ph.
SIAR	P101	Villaeles de Valdavia	-4.59	42.56	885	NR
SIAR	SA01	Ciudad Rodrigo	-6.54	40.59	635	Ph.
SIAR	SA02	Aldearrubia	-5.48	40.99	815	NR
SIAR	SA03	Aldearrubia	-5.48	40.99	815	Ph.
SIAR	SA101	Arabayona	-5.36	41.04	847	NR
SIAR	SA102	Ejeme	-5.53	40.78	816	NR
SIAR	SE01	Los Palacios y Villafranca	-5.94	37.18	21	NR
SIAR	SE02	Las Cabezas de San Juan	-5.88	37.02	25	Ph.
SIAR	SE03	Lebrija I	-6.13	36.98	25	Ph.
SIAR	SE05	Aznalcázar	-6.27	37.15	4	Ph.
SIAR	SE07	La Puebla del Río	-6.13	37.23	25	Ph.
SIAR	SE08	La Puebla del Río II	-6.05	37.08	41	Ph.
SIAR	SE09	Ecija	-5.08	37.59	125	Ph.
SIAR	SE10	La Luisiana	-5.23	37.53	188	Ph.
SIAR	SE101	IFAPA Finca Tomejil	-5.59	37.40	79	SC
SIAR	SE11	Osuna	-5.13	37.26	214	Ph.
SIAR	SE12	La Rinconada	-5.92	37.46	37	Ph.
SIAR	SE13	Sanlúcar la Mayor	-6.26	37.42	88	Ph.
SIAR	SE14	Villanueva Del Río y Minas	-5.68	37.61	38	NR
SIAR	SE15	Lora del Río	-5.54	37.66	68	Ph.
SIAR	SE16	Los Molares	-5.67	37.18	90	Ph.
SIAR	SE17	Guillena	-6.06	37.51	191	Ph.
SIAR	SE18	Puebla Cazalla	-5.35	37.22	229	Ph.
SIAR	SE19	IFAPA Centro las Torres-Tomejil	-5.96	37.51	11	Ph.
SIAR	SE20	Isla Mayor	-6.12	37.11	35	Ph.
SIAR	SE21	IFAPA Centro de los Palacios	-5.95	37.19	21	Ph.
SIAR	SE22	Villanueva Del Río y Minas	-5.69	37.59	30	Ph.
SIAR	SG01	Gomezserracín	-4.30	41.30	870	Ph.
SIAR	SG02	Nava de la Asunción	-4.48	41.17	822	Ph.
SIAR	SO01	Almazán	-2.50	41.46	943	Ph.
SIAR	SO02	San Esteban de Gormaz	-3.22	41.57	855	Ph.
SIAR	SO03	Fuentecantos	-2.43	41.83	1063	Ph.

SIAR	SO101	Hinojosa del Campo	-2.09	41.74	1043	NR
SIAR	TE01	Calanda	-0.21	40.96	433	Ph.
SIAR	TE02	Puig Moreno	-0.24	41.10	315	Ph.
SIAR	TE03	Híjar	-0.53	41.22	300	Ph.
SIAR	TE04	Monreal del Campo	-1.36	40.78	955	Ph.
SIAR	TE05	Teruel	-1.17	40.35	928	Ph.
SIAR	TE06	Villarquemado	-1.29	40.53	1004	Ph.
SIAR	TO01	Vegas de San Antonio	-4.70	39.96	400	Ph.
SIAR	TO03	Calera y Chozas	-4.97	39.87	387	NR
SIAR	TO04	Los Navalmorales	-4.66	39.72	725	Ph.
SIAR	TO05	Alcolea de Tajo	-5.14	39.81	371	Ph.
SIAR	TO06	Mora	-3.77	39.68	754	NR
SIAR	TO07	Recas	-3.98	40.06	609	Ph.
SIAR	TO08	Villarrubia de Santiago	-3.33	40.03	573	Ph.
SIAR	TO09	La Rinconada	-4.40	39.83	432	Ph.
SIAR	TO10	La Puebla de Almoradiel	-3.14	39.61	698	Ph.
SIAR	TO11	Magán	-3.94	39.94	507	Ph.
SIAR	TO12	Mora	-3.77	39.66	738	Ph.
SIAR	V01	Pedralba	-0.72	39.57	200	Ph.
SIAR	V02	Llíria	-0.63	39.69	250	Ph.
SIAR	V03	Turís	-0.69	39.40	299	Ph.
SIAR	V04	Benifaió	-0.46	39.28	72	Ph.
SIAR	V05	Cheste	-0.74	39.52	323	Ph.
SIAR	V06	Tavernes de Valldigna	-0.24	39.10	15	Ph.
SIAR	V07	Villanueva de Castellón	-0.52	39.07	58	Ph.
SIAR	V10	Benavites	-0.22	39.73	8	Ph.
SIAR	V101	Moncada Ivia	-0.40	39.59	35	SC
SIAR	V1010	Moncada Malla	-0.39	39.59	35	NR
SIAR	V102	Carcaixent Eea	-0.45	39.11	35	SC
SIAR	V1020	Carcaixent Eea Invernadero	-0.45	39.12	35	NR
SIAR	V103	Carlet Ce Coop	-0.55	39.23	35	SC
SIAR	V1030	Carlet Malla	-0.53	39.23	35	NR
SIAR	V104	Llutxent Eea	-0.36	38.94	200	SC
SIAR	V106	Requena Cerrito	-1.23	39.50	692	SC
SIAR	V107	Manises	-0.49	39.48	71	SC
SIAR	V14	Algemesí	-0.44	39.22	17	Ph.
SIAR	V16	Bétera	-0.47	39.60	117	NR
SIAR	V17	Picassent	-0.50	39.36	115	Ph.
SIAR	V18	Montesa	-0.64	38.95	267	Ph.
SIAR	V19	Xàtiva	-0.55	39.00	124	Ph.
SIAR	V20	Sagunt	-0.29	39.65	33	Ph.
SIAR	V21	Campo Arcís	-1.16	39.43	584	Ph.
SIAR	V22	Villalonga	-0.20	38.89	92	Ph.
SIAR	V23	Gandia Marxuquera	-0.25	38.96	113	Ph.
SIAR	V24	Polinyá de Xúquer	-0.36	39.19	4	NR
SIAR	V25	Bolbaite	-0.69	39.07	269	Ph.
SIAR	V26	Bétera	-0.47	39.60	95	Ph.
SIAR	V27	Chulilla	-0.83	39.68	380	Ph.
SIAR	VA01	Mayorga	-5.29	42.15	748	Ph.
SIAR	VA02	Torrecilla de la Orden	-5.21	41.23	793	Ph.
SIAR	VA03	Olmedo	-4.69	41.31	750	Ph.
SIAR	VA05	Encinas de Esgueva	-4.10	41.77	816	Ph.
SIAR	VA06	Tordesillas	-5.00	41.49	658	Ph.
SIAR	VA07	Valbuena de Duero	-4.27	41.64	756	Ph.
SIAR	VA08	Medina de Rioseco	-5.07	41.86	739	Ph.
SIAR	VA101	Finca Zamadueñas	-4.70	41.71	714	NR
SIAR	VA102	Medina del Campo	-4.90	41.31	726	NR
SIAR	VA103	Rueda	-4.98	41.40	709	NR
SIAR	Z01	Almonacid de la Sierra	-1.33	41.45	393	Ph.
SIAR	Z02	Belchite	-0.72	41.35	328	Ph.
SIAR	Z03	Quinto	-0.52	41.39	239	Ph.
SIAR	Z04	Fabara	0.15	41.17	246	Ph.
SIAR	Z05	Épila	-1.28	41.58	321	Ph.

SIAR	Z06	Ejea de los Caballeros	-1.20	42.10	317	Ph.
SIAR	Z07	Sádaba	-1.31	42.27	438	Ph.
SIAR	Z08	Luna	-0.94	42.10	409	Ph.
SIAR	Z09	Santa Engracia	-1.33	41.92	253	NR
SIAR	Z10	Pastriz	-0.75	41.60	192	NR
SIAR	Z11	Montañana	-0.82	41.71	222	Ph.
SIAR	Z13	Calatayud	-1.66	41.33	524	NR
SIAR	Z14	Borja	-1.51	41.86	366	Ph.
SIAR	Z15	Tarazona	-1.75	41.92	516	Ph.
SIAR	Z16	Caspe	-0.07	41.30	175	Ph.
SIAR	Z17	Osera de Ebro	-0.54	41.55	253	Ph.
SIAR	Z18	Daroca	-1.42	41.11	753	Ph.
SIAR	Z19	Zuera	-0.75	41.87	302	NR
SIAR	Z20	El Bayo	-1.25	42.18	379	Ph.
SIAR	Z21	Tauste	-1.14	42.00	361	Ph.
SIAR	Z22	Boquiñeni	-1.25	41.84	245	Ph.
SIAR	Z23	Pastriz	-0.73	41.59	192	Ph.
SIAR	Z24	Calatayud	-1.61	41.36	518	Ph.
SIAR	Z25	Santa Engracia (Tauste)	-1.31	41.91	250	Ph.
SIAR	Z26	Zuera	-0.77	41.89	282	Ph.
SIAR	Z27	Torres de Berrellén	-1.06	41.76	203	NR
SIAR	ZA01	Colinas de Trasmonte	-5.81	42.00	709	Ph.
SIAR	ZA02	Villaralbo	-5.65	41.48	659	Ph.
SIAR	ZA04	Villalpando	-5.39	41.88	701	Ph.
SIAR	ZA05	Pozuelo de Tábara	-5.90	41.78	714	Ph.
SIAR	ZA06	Barcial del Barco	-5.67	41.93	738	Ph.
SIAR	ZA07	Toro	-5.52	41.50	627	NR
SIAR	ZA08	Toro	-5.37	41.51	650	Ph.
Meteocat	C8	Cervera	1.30	41.68	554	FC
Meteocat	CD	La Seu Durgell	1.43	42.37	849	FC
Meteocat	CT	El Pont de Suert	0.74	42.40	823	FC
Meteocat	D4	Roses	3.18	42.27	24	FC
Meteocat	D9	El Vendrell	1.52	41.22	59	FC
Meteocat	DC	Olot – Pla de Baix	2.48	42.19	422	FC
Meteocat	DF	La Bisbal Demporda	3.04	41.98	29	FC
Meteocat	DQ	Vila-Rodona	1.36	41.31	287	FC
Meteocat	U7	Aldover	0.51	40.86	52	Ph.
Meteocat	UA	Asco	0.51	41.20	257	Ph.
Meteocat	UU	Amposta	0.63	40.71	3	FC
Meteocat	V8	El Poal	0.88	41.67	223	FC
Meteocat	VK	Raimat	0.45	41.68	286	Ph.
Meteocat	W6	Riudoms	1.02	41.14	158	Ph.
Meteocat	WA	Oliola	1.15	41.88	443	Ph.
Meteocat	WP	Canaletes	1.69	41.49	325	FC
Meteocat	WS	Viladrau	2.42	41.84	953	FC
Meteocat	X1	Falset	0.82	41.15	359	FC
Meteocat	X4	Barcelona – El Raval	2.17	41.38	33	FC
Meteocat	XE	Tarragona – Complex Educatiu	1.20	41.10	5	FC
Euskalmet	0AA	Etura	-2.50	42.89	549	SS
Euskalmet	0DC	Ibai Eder (Ibai Eder)	-2.26	43.17	90	SS
Euskalmet	0EC	Lasarte	-2.02	43.25	18	SS
Euskalmet	1	Arkaute I (Granja)	-2.63	42.85	517	SS
Euskalmet	1	Arkaute I (Granja)	-2.63	42.85	517	SS
Euskalmet	17	Miramon	-1.97	43.29	113	SS
Euskalmet	18	Higer	-1.80	43.39	43	SS
Euskalmet	19	Matxitxako (Repetidor)	-2.76	43.44	433	SS
Euskalmet	2	Arteaga	-2.66	43.35	19	SS
Euskalmet	20	Ventas De Armentia	-2.70	42.72	578	SS
Euskalmet	22	Urkiola (Puerto)	-2.65	43.10	709	SS
Euskalmet	23	Arrasate – Mondragón	-2.49	43.07	318	SS
Euskalmet	24	Iturrieta (Granja)	-2.35	42.79	987	SS
Euskalmet	26	Berastegi	-1.98	43.12	379	SS
Euskalmet	27	Llodio (Depuradora)	-2.95	43.14	207	SS

Euskalmet	29	Granja Fraisoro	-2.06	43.19	149	SS
Euskalmet	3	Derio (Granja)	-2.87	43.29	30	SS
Euskalmet	30	Salvatierra (Depuradora)	-2.40	42.86	589	SS
Euskalmet	33	Igorre (Deposito)	-2.78	43.17	150	SS
Euskalmet	34	Espejo	-3.04	42.81	504	SS
Euskalmet	35	Altube (Peaje)	-2.87	42.96	618	SS
Euskalmet	36	Iurreta	-2.62	43.17	175	SS
Euskalmet	39	Deusto	-2.97	43.28	3	SS
Euskalmet	40	Gasteiz (Lakua)	-2.69	42.86	546	SS
Euskalmet	42	Punta Galea (Faro)	-3.04	43.37	61	SS
Euskalmet	43	Ordizia (Oiangu)	-2.18	43.05	243	SS
Euskalmet	47	Kapildui (Repetidor)	-2.54	42.77	1173	SS
Euskalmet	48	Herrera (Puerto)	-2.68	42.60	1188	SS
Euskalmet	50	Zambrana (Peaje)	-2.89	42.67	470	SS
Euskalmet	51	Saratxo (Nerbioi)	-3.00	43.03	230	SS
Euskalmet	53	Barazar (Puerto)	-2.71	43.07	608	SS
Euskalmet	54	Otxandio	-2.66	43.04	556	SS
Euskalmet	55	Ozaeta (Barrundia)	-2.49	42.91	548	SS
Euskalmet	56	Alegria (Canal)	-2.52	42.84	545	SS
Euskalmet	57	Mungia (Depuradora)	-2.85	43.36	22	SS
Euskalmet	58	Bidania (Deposito)	-2.16	43.14	592	SS
Euskalmet	59	Ordunte (Embalse)	-3.28	43.16	300	SS
Euskalmet	60	Paganos	-2.60	42.56	577	SS
Euskalmet	61	Arboleda	-3.07	43.29	329	SS
Euskalmet	64	Zarautz (Camping)	-2.15	43.29	80	SS
Euskalmet	65	Cerroja-Karrantza	-3.41	43.21	677	SS
Euskalmet	69	Almike	-2.73	43.41	106	SS
Euskalmet	7	Santa Clara	-2.00	43.32	48	SS
Meteo Navarra	10	Yesa GN	-1.19	42.62	487	SS
Meteo Navarra	11	Villanueva de Yerri GN	-1.95	42.74	498	SS
Meteo Navarra	12	Beortegi GN	-1.43	42.80	580	SS
Meteo Navarra	22	Aralar GN	-1.96	42.95	1344	SS
Meteo Navarra	23	Arangoiti GN	-1.20	42.64	1353	SS
Meteo Navarra	24	Carrascal GN	-1.66	42.68	568	SS
Meteo Navarra	246	Getadar GN	-1.47	42.62	710	SS
Meteo Navarra	249	Erremendia (Salazar) GN	-1.19	42.88	1047	SS
Meteo Navarra	25	Gorramendi GN	-1.45	43.21	1071	SS
Meteo Navarra	250	Urbasa GN	-2.18	42.85	886	SS
Meteo Navarra	26	Bardenas (Loma Negra) GN	-1.38	42.07	647	SS
Meteo Navarra	28	El Perdon GN	-1.71	42.73	1024	SS
Meteo Navarra	29	Trinidad De Iturgoien GN	-1.98	42.81	1224	SS
Meteo Navarra	30	Ujue GN	-1.51	42.51	829	SS
Meteo Navarra	31	Bardenas (El Yugo) GN	-1.58	42.20	486	SS
Meteo Navarra	33	Aguilar De Codes GN	-2.40	42.61	736	SS
Meteo Navarra	34	Aoiz GN	-1.37	42.79	534	SS
Meteo Navarra	35	Carcastillo (La Oliva) GN	-1.46	42.37	343	SS
Meteo Navarra	36	Tudela (Montes Del Cierzo) GN	-1.65	42.13	314	SS
Meteo Navarra	37	Oskotz GN	-1.76	42.95	562	SS
Meteo Navarra	405	Pamplona (ESTIA) UPNA	-1.63	42.79	433	SS
Meteo Navarra	42	Doneztebe-Santesteban GN	-1.66	43.13	125	SS
Meteo Navarra	455	Pamplona GN	-1.64	42.82	455	SS
Meteo Navarra	7	Estella GN	-2.03	42.67	486	SS
Meteo Navarra	8	Etxarri-Aranatz GN	-2.06	42.91	505	SS
Meteo Navarra	9	Tafalla GN	-1.69	42.52	430	SS
MeteoGalicia	10045	Mabegondo	-8.26	43.24	94	FC
MeteoGalicia	10046	Marco da Curra	-7.89	43.34	651	FC
MeteoGalicia	10047	Pedro Murias	-7.08	43.54	51	FC
MeteoGalicia	10048	O Invernadeiro	-7.34	42.12	1026	FC
MeteoGalicia	10049	Corrubedo	-9.03	42.56	30	SC
MeteoGalicia	10050	Cis Ferrol	-8.25	43.49	37	FC
MeteoGalicia	10052	Muralla	-8.77	42.75	661	Ph.
MeteoGalicia	10053	Campus Lugo	-7.55	42.99	400	FC
MeteoGalicia	10055	Guitiriz-Mirador	-7.78	43.23	684	SC

MeteoGalicia	10056	Marroxo	-7.50	42.47	645	Ph.
MeteoGalicia	10057	Alto do Rodicio	-7.59	42.30	981	FC
MeteoGalicia	10058	Verín-Vilamaior	-7.40	41.98	546	Ph.
MeteoGalicia	10060	Monte Aloia	-8.68	42.08	484	SC
MeteoGalicia	10061	Mouriscade	-8.14	42.62	500	FC
MeteoGalicia	10062	Ancares	-6.92	42.82	1364	FC
MeteoGalicia	10063	Queimadelos	-8.43	42.23	371	FC
MeteoGalicia	10064	Lourizán	-8.66	42.41	57	FC
MeteoGalicia	10067	Castrove	-8.70	42.46	424	Ph.
MeteoGalicia	10085	Corón	-8.80	42.58	3	SC
MeteoGalicia	10086	Fornelos de Montes	-8.40	42.32	705	Ph.
MeteoGalicia	10087	Fontecada	-8.87	42.97	369	FC
MeteoGalicia	10088	Fragavella	-7.44	43.46	595	FC
MeteoGalicia	10089	Melide	-7.98	42.91	447	Ph.
MeteoGalicia	10091	Castro Vicaludo	-8.86	42.00	473	Ph.
MeteoGalicia	10092	Punta Candieira	-8.05	43.71	254	FC
MeteoGalicia	10093	Malpica	-8.83	43.34	161	SC
MeteoGalicia	10094	Olas	-8.28	43.13	401	Ph.
MeteoGalicia	10095	Sergude	-8.46	42.82	231	Ph.
MeteoGalicia	10096	Río do Sol	-8.69	43.10	540	Ph.
MeteoGalicia	10097	Serra da Faladoira	-7.79	43.59	576	SC
MeteoGalicia	10098	Abradelo	-7.25	42.75	826	SC
MeteoGalicia	10099	Bóveda	-7.47	42.65	432	FC
MeteoGalicia	10100	Conchada	-7.27	42.49	697	NR
MeteoGalicia	10101	Corno do Boi	-7.89	43.04	731	Ph.
MeteoGalicia	10102	Courel	-7.19	42.60	777	Ph.
MeteoGalicia	10103	Foz	-7.28	43.56	73	SC
MeteoGalicia	10104	Penedo do Galo	-7.56	43.66	545	SC
MeteoGalicia	10105	Pol	-7.28	43.16	647	FC
MeteoGalicia	10106	A Pontenova	-7.17	43.34	490	Ph.
MeteoGalicia	10107	Sambreixo	-7.79	43.15	496	FC
MeteoGalicia	10108	Portomarín	-7.62	42.81	447	Ph.
MeteoGalicia	10109	Amiudal	-8.24	42.42	553	SC
MeteoGalicia	10110	Baltar	-7.71	41.95	807	SC
MeteoGalicia	10111	Entrimo	-8.10	41.95	763	SC
MeteoGalicia	10112	Gandarela	-7.97	42.17	623	FC
MeteoGalicia	10113	Monte Medo	-7.63	42.23	608	Ph.
MeteoGalicia	10114	As Petarelas	-6.93	42.46	577	FC
MeteoGalicia	10115	Serra do Eixe	-7.01	42.36	1229	Ph.
MeteoGalicia	10116	Viana do Bolo	-7.09	42.16	851	Ph.
MeteoGalicia	10117	San Xoán de Río	-7.30	42.40	1026	Ph.
MeteoGalicia	10118	Burela	-7.37	43.65	421	SC
MeteoGalicia	10119	Xurés	-7.97	41.90	1059	SC
MeteoGalicia	10120	Pereira	-8.32	42.63	717	SC
MeteoGalicia	10121	Rebordelo	-8.50	42.47	367	SC
MeteoGalicia	10122	Serra Do Faro	-7.93	42.58	991	SC
MeteoGalicia	10124	Santiago-Eoas	-8.56	42.88	255	FC
MeteoGalicia	10125	Illas Cíes	-8.90	42.22	25	SC
MeteoGalicia	10126	Ons	-8.93	42.38	121	SC
MeteoGalicia	10127	Caldas de Reis	-8.62	42.60	268	SC
MeteoGalicia	10128	Sálvora	-9.01	42.47	24	Ph.
MeteoGalicia	10129	Sanxenxo	-8.80	42.40	34	Ph.
MeteoGalicia	10130	Lardeira	-6.78	42.38	1620	SC
MeteoGalicia	10131	Cabeza de Manzaneda	-7.30	42.26	1758	SC
MeteoGalicia	10132	O Cebreiro	-7.05	42.71	1310	SC
MeteoGalicia	10135	Lira	-9.12	42.80	170	SC
MeteoGalicia	10136	O Xipro	-7.05	43.18	789	SC
MeteoGalicia	10137	Ventosa	-6.91	42.96	910	SC
MeteoGalicia	10138	Xares	-6.89	42.21	1762	SC
MeteoGalicia	10141	Aldea Nova	-8.13	43.56	278	Ph.
MeteoGalicia	10143	Cariño	-7.86	43.74	5	SC
MeteoGalicia	10144	Arzúa	-8.17	42.93	362	SC
MeteoGalicia	10146	Castro R. de Lea	-7.48	43.16	428	Ph.

MeteoGalicia	10153	A Gándara	-9.06	43.11	405	SC
MeteoGalicia	10154	O Viso	-8.60	42.32	260	SC
MeteoGalicia	10161	Vigo-Campus	-8.68	42.17	460	FC
MeteoGalicia	10162	Borreiros	-7.63	43.63	59	Ph.
MeteoGalicia	10500	Verín-Vilela	-7.44	41.95	392	SC
MeteoGalicia	10800	Camariñas	-9.18	43.13	5	SC
MeteoGalicia	19065	Pé Redondo	-8.73	42.51	150	Ph.
MeteoGalicia	19066	Barrantes	-8.77	42.51	17	Ph.
MeteoGalicia	19068	Simes	-8.77	42.44	97	Ph.
MeteoGalicia	19069	A Lanzada	-8.87	42.46	9	Ph.
MeteoGalicia	19070	A Armenteira	-8.74	42.47	256	SC
MeteoGalicia	50500	Santiago-San Lázaro	-8.52	42.80	305	SS
SIAR Rioja	501	Agoncillo	-2.29	42.47	342	SC
SIAR Rioja	502	Aldeanueva De Ebro	-1.90	42.22	365	SC
SIAR Rioja	503	Sto.domingo De La Calzada	-2.94	42.43	640	SC
SIAR Rioja	504	Villar De Torre	-2.86	42.38	727	SC
SIAR Rioja	505	Casalarreina	-2.90	42.54	510	SC
SIAR Rioja	506	Alfaro	-1.78	42.15	315	SC
SIAR Rioja	507	Uruñuela - Torremontalbo	-2.71	42.46	465	SC
SIAR Rioja	508	Rincón De Soto	-1.85	42.25	270	SC
SIAR Rioja	509	Logroño	-2.51	42.44	465	SC
SIAR Rioja	510	San Vicente De La Sonsierra	-2.73	42.57	543	SC
SIAR Rioja	511	Quel (Antes Autol)	-2.04	42.25	430	SC
SIAR Rioja	512	Pazuengos	-2.91	42.34	1299	SC
SIAR Rioja	513	Leiva	-3.05	42.50	595	SC
SIAR Rioja	514	Cervera (Cabretón)	-1.89	42.01	495	SC
SIAR Rioja	515	Igea	-1.99	42.06	564	SC
SIAR Rioja	516	Foncea	-3.04	42.61	669	SC
SIAR Rioja	517	Calahorra	-2.00	42.33	328	SC
SIAR Rioja	518	Ausejo	-2.15	42.34	537	SC
SIAR Rioja	519	Albelda De Iregua	-2.47	42.38	487	SC
SIAR Rioja	520	Arenzana De Abajo	-2.72	42.39	523	SC
SIAR Rioja	521	Santa Engracia Del Jubera	-2.26	42.37	570	SC
SOS Rioja	1	Ezcaray	-3.01	42.33	1000	FC
SOS Rioja	10	Urbaña	-2.85	42.17	1565	FC
SOS Rioja	11	Moncalvillo	-2.62	42.33	1495	FC
SOS Rioja	1183	Santa Marina	-2.37	42.24	1285	NR
SOS Rioja	12	Aguilar	-1.97	41.97	752	FC
SOS Rioja	13	Calahorra	-2.00	42.29	350	FC
SOS Rioja	1352	Alfaro	-1.74	42.18	374	FC
SOS Rioja	2	Haro	-2.84	42.57	460	NR
SOS Rioja	3	Arnedo	-2.09	42.23	545	NR
SOS Rioja	4	Nájera	-2.72	42.42	510	NR
SOS Rioja	5	Villoslada	-2.67	42.12	1235	FC
SOS Rioja	6	San Román	-2.46	42.23	1094	FC
SOS Rioja	7	Ocón	-2.23	42.29	1105	FC
SOS Rioja	8	Yerga	-1.97	42.14	1101	FC
SOS Rioja	888	Torrecilla	-2.62	42.25	938	FC
SOS Rioja	9	Logroño	-2.47	42.46	408	FC
